# Functional expression of opioid receptors and other human GPCRs in yeast engineered to produce human sterols

**DOI:** 10.1038/s41467-022-30570-7

**Published:** 2022-05-24

**Authors:** Björn D. M. Bean, Colleen J. Mulvihill, Riddhiman K. Garge, Daniel R. Boutz, Olivier Rousseau, Brendan M. Floyd, William Cheney, Elizabeth C. Gardner, Andrew D. Ellington, Edward M. Marcotte, Jimmy D. Gollihar, Malcolm Whiteway, Vincent J. J. Martin

**Affiliations:** 1 Department of Biology, Centre for Applied Synthetic Biology, Concordia University, Montréal, QC H4B1R6 Canada; 2 Department of Molecular Biosciences, Center for Systems and Synthetic Biology, The University of Texas at Austin, Austin, TX 78712 USA; 3 DEVCOM Army Research Laboratory-South, Austin, 78712 TX USA; 4 Center for Molecular and Translational Human Infectious Diseases Research, Department of Pathology and Genomic Medicine, Houston Methodist Research Institute, Houston Methodist Hospital, Houston, TX USA

**Keywords:** Synthetic biology, Sterols

## Abstract

The yeast *Saccharomyces cerevisiae* is powerful for studying human G protein-coupled receptors as they can be coupled to its mating pathway. However, some receptors, including the mu opioid receptor, are non-functional, which may be due to the presence of the fungal sterol ergosterol instead of cholesterol. Here we engineer yeast to produce cholesterol and introduce diverse mu, delta, and kappa opioid receptors to create sensitive opioid biosensors that recapitulate agonist binding profiles and antagonist inhibition. Additionally, human mu opioid receptor variants, including those with clinical relevance, largely display expected phenotypes. By testing mu opioid receptor-based biosensors with systematically adjusted cholesterol biosynthetic intermediates, we relate sterol profiles to biosensor sensitivity. Finally, we apply sterol-modified backgrounds to other human receptors revealing sterol influence in SSTR5, 5-HTR4, FPR1, and NPY1R signaling. This work provides a platform for generating human G protein-coupled receptor-based biosensors, facilitating receptor deorphanization and high-throughput screening of receptors and effectors.

## Abstract (web summary)

The yeast *Saccharomyces cerevisiae* is powerful for studying human G protein-coupled receptors as they can be coupled to its mating pathway. Here the authors engineer baker’s yeast to produce human sterols and show that vertebrate G protein coupled receptors are more sensitive in this membrane environment.

## Introduction

G-protein coupled receptors (GPCRs) detect diverse extracellular stimuli, modulating signal transduction pathways that allow cells to respond to their environment. These seven transmembrane domain proteins typically function by binding external ligands, which induce conformational changes, propagating a signal across the plasma membrane and triggering internal signaling pathways^1^. Owing to their critical functions and their ubiquity as the largest family of human membrane proteins, one-third of current FDA-approved therapeutic targets are GPCRs^2^. Yet, while these targets are functionally understood, discovery of new GPCR-interacting therapeutics remains challenging in part due to screening limitations.

Assays of GPCR activity in the yeast *S. cerevisiae* may accelerate the search for therapeutics by allowing simple, cheap, and high-throughput screens. Commonly, these assays are based on functionally linking GPCRs to the yeast pheromone response pathway (PRP). Normally in the PRP, a native GPCR binds a mating pheromone, causing a GTP-GDP substitution in the Gɑ protein Gpa1, triggering a mitogen-activated protein kinase signaling cascade culminating in upregulation of Ste12-regulated genes^3,4^. This pathway can be commandeered to make a biosensor by replacing the native GPCR, creating a chimeric Gpa1 to maintain the GPCR-G protein interaction, and placing a reporter under the control of a Ste12-regulated promoter^5^. Such yeast-based biosensor designs, initially applied to the β2-adrenergic receptor^6^, have now been used for over 50 receptors^7^. Yet, in many cases, GPCRs cannot be functionally expressed in yeast^8–10^. This may be due to poor expression or folding^11–13^, defects in trafficking to the plasma membrane^10^, or differences in the chemical environment^14–16^.

In particular, the function of heterologously-expressed human GPCRs may be disrupted by the membrane lipid composition in yeast, as the dominant sterol is ergosterol, as opposed to cholesterol^9^. This would be consistent with past work documenting the importance of cholesterol-GPCR interactions^17–19^ and the frequent presence of cholesterol molecules as elements of established GPCR structures^20^. Thus, modifying the sterol profile of yeast may increase the proportion of human GPCRs that can be functionally expressed. Previously, deletion of the ergosterol biosynthetic genes *ERG5/6*, and introduction of zebrafish enzymes DHCR7/24 resulted in yeast producing cholesterol up to 96% of total sterol content^21,22^. While this modification disrupted the function of the endogenous GPCR Ste2^21^, its effect on heterologous GPCRs has not been tested.

It would be valuable to apply yeast-based rapid screening approaches to human opioid receptors. The main opioid receptor types, mu, delta, and kappa, are all GPCRs implicated in nociception and analgesia^23^. Drugs targeting these receptors, and the human mu opioid receptor (HsMOR) in particular, are essential front-line pain treatment medicines, but have also enabled misuse and dependence^24^. Expansion of available drugs that target these receptors but lack the side-effects of prototypical opioids could help resolve these issues. Though an HsMOR-based biosensor would provide a powerful tool for identifying new drug candidates, past construction efforts have failed in part due to the sterol composition of yeast membranes leading to low HsMOR agonist binding^9^.

Here we describe a biosensor background based on signaling through the PRP in a yeast strain engineered to produce cholesterol. This background dramatically improves HsMOR activity relative to an ergosterol-rich strain, enabling the characterization of structural and clinically-relevant HsMOR variants. We probed the agonist sensitivities of opioid biosensors based on 15 different receptors and found that opioid receptor activity and agonist specificities are well conserved in yeast. Screening a library of HsMOR-based biosensors with different sterol profiles allowed us to uncover how cholesterol intermediates affect signaling and establish that the cholesterol-producing background was highly effective. Lastly, we applied the cholesterol-producing background as a platform to study other human GPCRs.

## Results

### Construction of an opioid biosensor in a cholesterol-producing yeast

Previous work found that yeast-expressed human mu opioid receptor (HsMOR) was only able to bind agonists in lysates when ergosterol was removed and cholesterol added^9^. Therefore, we investigated whether HsMOR may be active in yeast cells engineered to produce cholesterol instead of ergosterol, and if active, whether linking HsMOR to the PRP would create an opioid biosensor.

**Fig. 1:**
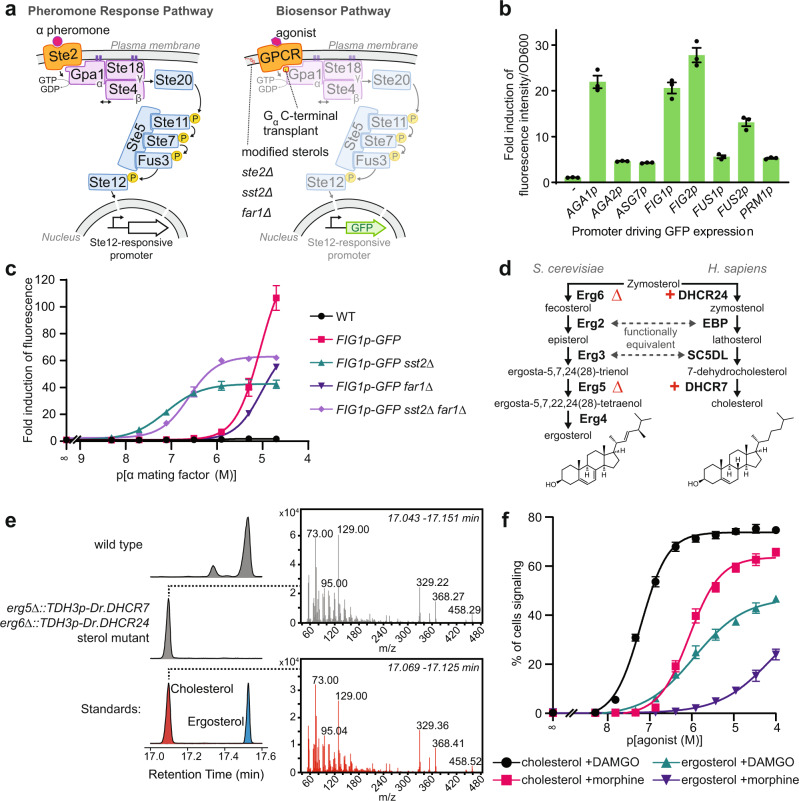
Development of a yeast-based opioid biosensor. **a** Strategy to adapt the yeast pheromone response pathway (PRP, left) into a biosensor pathway (right). An exogenous GPCR is introduced and linked to the pathway by a Gpa1 chimera. Deletion of *STE2*, *SST2*, and *FAR1* respectively prevents interference, potentiates signaling, and blocks signaling-induced cell cycle arrest. Ergosterol is replaced by cholesterol and a promoter controlled by the PRP-responsive Ste12 transcription factor drives GFP expression. **b** Biosensor reporter promoters tested using alpha mating factor-induced GFP expression from yeast Ste12-responsive promoters measured on a plate reader after 3 h treatment. *n* = 3 biologically independent experiments. **c** PRP sensitivity and activity resulting from *SST2* and/or *FAR1* deletions. Strains were treated with alpha mating factor for 6 h and mean fluorescence was measured by flow cytometry. *n* = 3 biologically independent experiments; 10,000 cells/strain/replicate. **d** Ergosterol and cholesterol biosynthetic pathways from zymosterol. **e** GC-MS analysis of sterol extracts showing successful synthesis of cholesterol in an *erg5/6 DHCR7/24* background. Chromatograms indicating retention times of derivatized sterols from a wild type strain (top), the engineered strain (middle), and standards (bottom). MS spectra extracted from engineered strain (top) and cholesterol standard (bottom). **f** DAMGO and morphine dose-response curves for ergosterol- and cholesterol-producing HsMOR-based biosensors. Measured by flow cytometry after 8 h agonist treatment. *n* = 3 biologically independent experiments; 10,000 cells/strain/replicate. Data presented as mean +/− SEM. Source data are provided as a Source Data file.

**Fig. 2:**
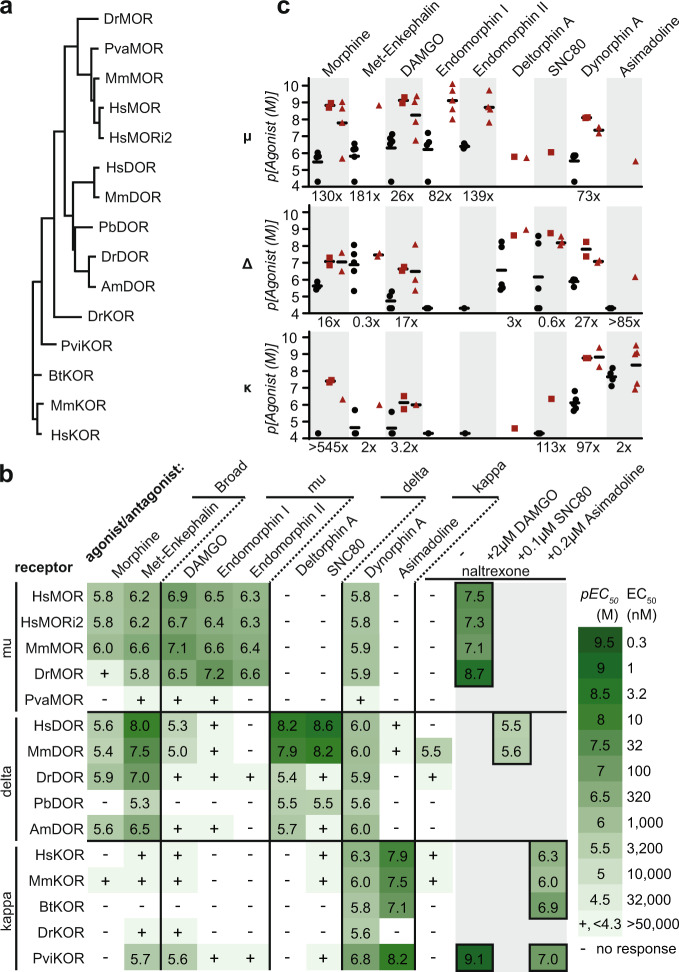
Activity of opioid receptors in the cholesterol-producing strain. **a** A protein-based phylogenetic tree of opioid receptors selected to make biosensors. MOR, mu receptor, KOR, kappa receptor, DOR, delta receptor. Dr, *D. rerio*, zebrafish; Pva, *P. vampyrus*, flying fox; Mm, *M. musculus*, mouse; Pb, *P. bivittatus*, python; Am, *A. mexicanus*, Mexican tetra; Pvi, *P. vitticeps*, bearded dragon; Bt, *B. taurus*, cow. i2, isoform 2. **b** Activities of agonists and the antagonist naltrexone on opioid receptors were assayed by flow cytometry and mean *pEC*_*50*_ was determined. Agonists clustered by literature receptor-type specificity. *n* = 3 biologically independent experiments, >2073 cells/condition/replicate. **c** Biosensor agonist sensitivities (EC_50_, black circles) relative to sensitivities reported in the literature for mammalian receptors as inhibition constants (Ki, red squares) and EC_50_/IC_50_ values (red triangles)^34–45^. Fold decrease in sensitivity of the best biosensor relative to the literature average is indicated. Source data are provided as a Source Data file.

We made a biosensor chassis based on previous studies linking GPCRs to the PRP^5,25^ (Fig. 1a). The pheromone receptor, Ste2, was removed to avoid interference and the final five residues of the Gɑ protein, Gpa1, were swapped with the endogenous HsMOR-interacting protein G_iα3_ (K_468_IGII > ECGLY) to generate a chimera previously shown to link exogenous GPCRs to the PRP^5^. We chose green fluorescent protein (GFP) expression as an output and selected the promoter controlling expression by using alpha mating factor to screen the ability of eight highly PRP-regulated promoters to express GFP in a wild type background^26,27^ (Fig. 1b). While *FUS1p* is often used^5,6,28^, we found that *AGA1p*, *FIG1p,* and *FIG2p* all yield roughly four times the response, leading us to select *FIG1p::GFP* as the reporter.

The chassis was optimized by deleting *FAR1* and *SST2*, respectively preventing PRP-induced cell cycle arrest and increasing sensitivity by reducing pathway deactivation. While these deletions are a common strategy^7^, their effects on heterologous signaling are poorly documented, so we measured how they influenced *FIG1p::GFP* response to alpha mating factor (Fig. 1c; Supplementary Fig. 1A). As expected, *SST2* deletion increased sensitivity (17.9×) though background fluorescence also increased (4.2×), which limited fold induction of fluorescence. While Far1 is not prescribed a role in pheromone sensitivity we found that *FAR1* deletion decreased sensitivity both in wild type and *sst2* backgrounds (7.0× and 3.1× respectively). The background fluorescence of the *sst2* strain was also reduced by *FAR1* deletion from 4.2× to 1.6× that of wild type. This, together with the ability of a *far1* strain to facilitate longer assays, led us to select a *sst2 far1* background even though the *FAR1* deletion impacts sensitivity.

Next, the strain was engineered to produce cholesterol instead of ergosterol. Cholesterol and ergosterol are structurally similar, with zymosterol as the last common intermediate (Fig. 1d). Following Souza et al., we deleted *ERG5/6* and added *TDH3p*-driven zebrafish *DHCR7/24* to block ergosterol production and redirect zymosterol to cholesterol^22^. In this modified cholesterol production pathway Erg2 and Erg3 fulfill the roles of human EBP and SC5DL respectively. GC-MS analysis showed 94% of sterols were cholesterol with 4% dehydrolathosterol also present (Figs. 1e, 6b; Supplementary Fig 4).

Addition of yeast codon-optimized *OPRM1*, the gene encoding HsMOR, driven by the strong *CCW12* promoter resulted in a candidate opioid biosensor. The sensitivities of both this cholesterol membrane biosensor, and a native yeast membrane (ergosterol) biosensor, were assessed by measuring fluorescence after 8 h exposure to different concentrations of the HsMOR agonists [D-Ala^2^, N-MePhe^4^, Gly-ol]-enkephalin (DAMGO) and morphine. Importantly, initial tests indicated a strong pH dependence, with optimal morphine signaling at pH 7.1, as opposed to the normal yeast growth media pH of 5-5.5 (Supplementary Fig 1B). We postulate that improved biosensor signaling at a pH of 7.1 results from conditions that more closely resemble the conditions HsMOR is exposed to in the brain (pH 7.2 intracellular^29^, pH 7.4 extracellular^30^). Furthermore, we found that monitoring the percent of cells signaling, defined as the percent of cells fluorescing above a background threshold, yielded the most sensitivity and was therefore used in all subsequent analyses (Supplementary Fig. 1C, D).

With pH adjustment, both ergosterol- and cholesterol-producing biosensors responded to DAMGO and morphine (Fig. 1f). Consistent with the known cholesterol dependence of HsMOR^9^, the cholesterol-rich biosensor was dramatically more effective, with a lower EC_50_ (62 ± 3 nM vs 1.3 ± 0.3 μM DAMGO; 0.9 ± 0.1 μM vs ~110 ± 40 μM morphine) and a larger proportion of cells signaling. The presence of any signaling in the ergosterol strain was unexpected given that previously [^3^H]DAMGO binding by HsMOR had not been detected in yeast^9^. The absence of binding may have been due to the use of a higher buffer pH (7.5) and/or lower receptor expression. Taken together, we have constructed two opioid biosensors with different detection limits that demonstrate conversion of sterols to cholesterol can improve human GPCR function in yeast.

### An array of biosensors based on different opioid receptors reveals fidelity of agonist selectivity

Next, we expanded the set of receptors being tested to explore the degree of opioid receptor functional conservation in yeast. We selected a diverse group, including five of each type: mu (MOR), kappa (KOR), and delta (DOR). Given that opioid receptors exist throughout Vertebrata, receptors from humans (*Homo sapiens*, Hs), mice (*Mus musculus*, Mm), and zebrafish (*Danio rerio*, Dr) were selected. Additional receptors were included from the cow (*Bos taurus*, Bt), flying fox (*Pteropus vampyrus*, Pva), bearded dragon (*Pogona vitticeps*, Pvi), Burmese python (*Python bivittatus*, Pb), and Mexican tetra, (*Astyanax mexicanus*, Am). As expected from the high degree of opioid receptor conservation, a MUSCLE-generated^31^ phylogenetic tree showed segregation by receptor type (Fig. 2a). Furthermore, MORs and DORs clustered closely, consistent with the current model of MORs and DORs emerging from a common ancestral receptor^32^.

Biosensors based on these opioid receptors were tested for activity and agonist specificity. Agonists were selected based on human receptor specificities: morphine and met-enkephalin are broad-acting^33^, DAMGO and Endomorphins I/II are MOR-specific^34,35^, Deltorphin A and SNC80 are DOR-specific^36,37^, and Dynorphin A and Asimadoline are KOR-specific^38,39^. Most were short 4–17 residue peptides, or peptide-based, with the exception of the benzylisoquinoline alkaloid morphine and the two heterocycles SNC80 and Asimadoline. For each biosensor-agonist pair, a dose-response curve was made and EC_50_ was calculated (Fig. 2b and Supplementary Figs. 1, 2). Remarkably, all strains showed some response to at least three of the agonists tested. Furthermore, with the exception of the PvaMOR strain, all biosensors were sensitive enough to use agonist concentrations below 50 μM to determine EC_50_, and when signaling was sufficient for EC_50_ determination the average fold induction of fluorescence was 18× (Supplementary Data 6).

Agonist specificities largely matched those of human receptors in endogenous conditions, though receptor sensitivity was reduced (Fig. 2b, c). MORs and DORs responded to the broad acting agonists morphine and met-enkephalin with EC_50_s of 30 nM–3 μM, while KORs responded poorly, consistent with KORs’ reported low met-enkephalin sensitivity but not their reported 47–538 nM morphine sensitivity^34,35^. MOR-specific agonists were detected by MORs with 60–500 nM EC_50_s while other receptor types were less sensitive (EC_50_ > 5 μM). Likewise, only DORs fully responded to the HsDOR-specific agonists deltorphin A and SNC80 (EC_50_s 2.5 nM–4 μM). KOR-based biosensors were most sensitive to KOR-specific agonists with EC_50_s as low as 6.3 nM, though most biosensors responded to Dynorphin A, consistent with reported MOR and DOR Dynorphin A sensitivity^34^. While agonist specificities were maintained, biosensors displayed type-specific decreases in receptor sensitivity relative to values reported for more native environments, with DORs performing best (11× decrease) followed by KORs (43× decrease) and MORs (105× decrease)^34–45^ (Fig. 2c and Supplementary Data 5).

The effects of an antagonist, naltrexone, were also determined^34^. Alone, naltrexone occasionally functioned as a partial agonist, at most eliciting a signaling population one seventh the size of that induced by a strong agonist (Fig. 2b and Supplementary Fig 1G). Antagonist activity was tested by incubating biosensors for eight hours with an amount of agonist sufficient for strong signaling (2 μM DAMGO, 0.1 μM SNC80, 0.2 μM asimadoline) and varying concentrations of naltrexone (Fig. 2b and Supplementary Fig 1G). Naltrexone blocked activity in all cases and, in line with binding coefficients previously determined in CHO cells^34^, MORs were most sensitive (IC_50_: 2–80 nM), followed by KORs (IC_50_: 0.8–500 nM) and DORs (IC_50_: 2.5–3.2 μM). Together, the ability of these biosensors to reconstitute both agonist specificities and antagonist activity make them powerful tools for assessing how opioid receptors in native environments will respond to a compound.

### Signal sequences disrupt mu opioid receptor function

**Fig. 3:**
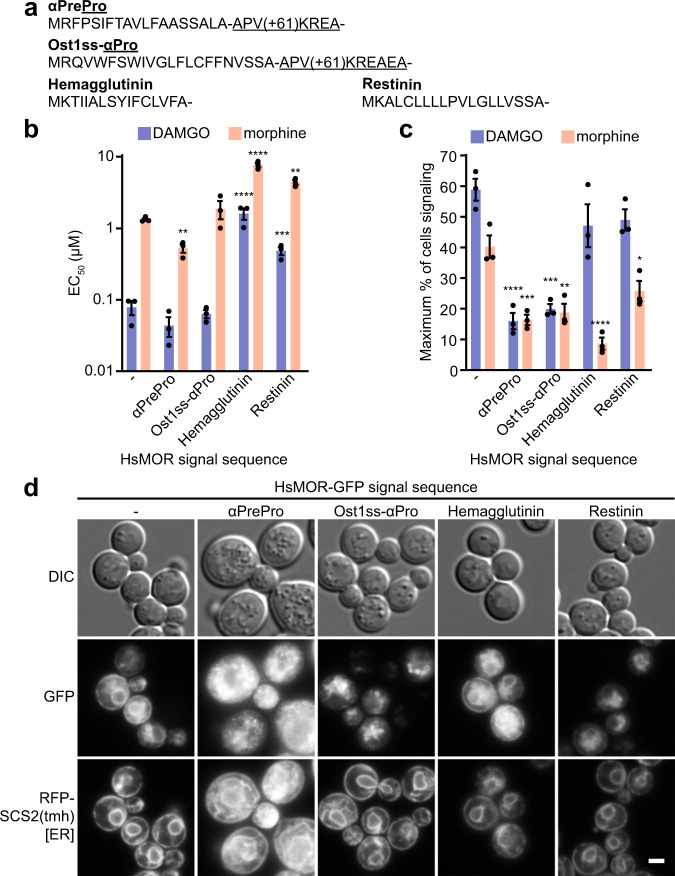
Effect of signal sequences on HsMOR function. **a** N-terminal signal sequences tested on HsMOR. **b** Effect of signal sequences on HsMOR biosensor sensitivity to DAMGO and morphine. Unpaired one-way ANOVA of *pEC*_*50*_s: *n* = 3 biologically independent experiments, >7595 cells/strain/replicate; *P* < 0.0001 for each agonist; Dunnett’s multiple comparisons tests against WT shown. **c** Effect of signal sequences on HsMOR signaling population. Unpaired one-way ANOVA: *n* = 3 biologically independent experiments, >7595 cells/strain/replicate; *P* < 0.0001 (DAMGO) and *P* < 0.001 (morphine); Dunnett’s multiple comparisons tests against WT shown. **d** Imaging of HsMOR-GFP with the indicated signal sequences in a cholesterol-rich background with the ER marker RFP-SCS2(tmh). *n* = 3 biologically independent experiments. Data presented as mean +/− SEM. Scale bar is 2 μm. *, *P* < 0.05; **, *P* < 0.01; ***, *P* < 0.001; ****, *P* < 0.0001. Source data are provided as a Source Data file.

Although the biosensors recapitulated the pattern of response seen in vertebrates, sensitivity was lower than in native cells, suggesting aspects of receptor expression or the signaling environment could be improved. To explore if opioid receptor sensitivity was limited by expression or localization defects, GFP-tagged HsMOR (HsMOR-GFP) was imaged in a cholesterol-producing background. HsMOR-GFP primarily localized to the ER with a secondary vacuolar pool (Fig. 3d). The unexpected lack of HsMOR-GFP on the plasma membrane, where functional GPCRs have previously been observed^10^, suggests a folding or trafficking defect may be leading to endoplasmic reticulum (ER) retention and/or misdirection to the vacuole. GFP tagging itself is unlikely to be causing mislocalization as the tag only reduced biosensor response to DAMGO by 34% (Supplementary Fig. 1E, F). Given the degree of HsMOR-GFP ER retention, we speculated that increasing plasma membrane localization might improve biosensor function.

GPCR expression and localization can be improved by appending N-terminal signal sequences^10,46^, short peptides that mediate ER insertion^47^. While integral membrane proteins, such as opioid receptors, often lack signal sequences because transmembrane helices are sufficient for ER targeting, adding the sequences can increase ER insertion speed, minimizing misfolding^47,48^. Therefore, the effects of appending signal sequences to HsMOR were assessed. We tested sequences from yeast (α-mating factor pre-pro, αPrePro; Ost1 signal peptide - α-mating factor pro, Ost1ss-αPro) as well as others previously used to improve GPCR expression in mammalian cells (influenza Hemagglutinin; Restinin)^46,49^ (Fig. 3a).

Appending signal sequences to HsMOR generally did not improve sensitivity to either DAMGO or morphine, and was instead disruptive in two distinct ways (Fig. 3b, c and Supplementary Fig. 3A, B). Only αPrePro increased sensitivity, by roughly two-fold for both agonists, whereas Ost1ss-αPro was neutral and the Hemagglutinin and Restinin sequences caused 3-20-fold decreases in sensitivity. While the yeast αPrePro and Ost1ss-αPro sequences were neutral or beneficial for sensitivity, they dramatically decreased the maximum size of the signaling population, by 72 and 66% respectively. In contrast, the Hemagglutinin and Restinin sequences didn’t significantly affect the DAMGO-induced signaling population, selectively disrupting the morphine response. Taken together, the signal sequence classes have contrasting effects: yeast-based sequences had a neutral or positive effect on sensitivity and a reduced signaling population, whereas the Hemagglutinin and Restinin sequences disrupted sensitivity but did not always reduce the signaling population.

To better understand this dichotomy, signal sequence-tagged HsMOR-GFP was imaged in a cholesterol-producing background (Fig. 3d). Strikingly, the αPrePro and Ost1ss-αPro sequences resulted in enlarged granular cells, expanded ER membranes, and relocalization of HsMOR-GFP to puncta. In contrast, the Hemagglutinin and Restinin tags did not disrupt cellular morphology and HsMOR-GFP remained ER-localized, though the vacuolar pool may have increased. These results suggest that the yeast-based sequences cause global cellular disruptions, perhaps through partial HsMOR-GFP misfolding, which may be associated with premature ER exit. Cellular stress likely disrupts signaling, leading to the observed reductions in biosensor signaling competency. The other sequences did not disrupt cellular morphology and consequently did not display consistent decreases in the biosensor signaling population. The link between cellular localization and sensitivity was unclear. Overall, while the yeast signal sequences subtly improved HsMOR sensitivity, the associated cellular disruptions decreased the signaling population such that the signal sequences were not beneficial.

### Biosensors recapitulate the effects of missense mutants in HsMOR

**Fig. 4:**
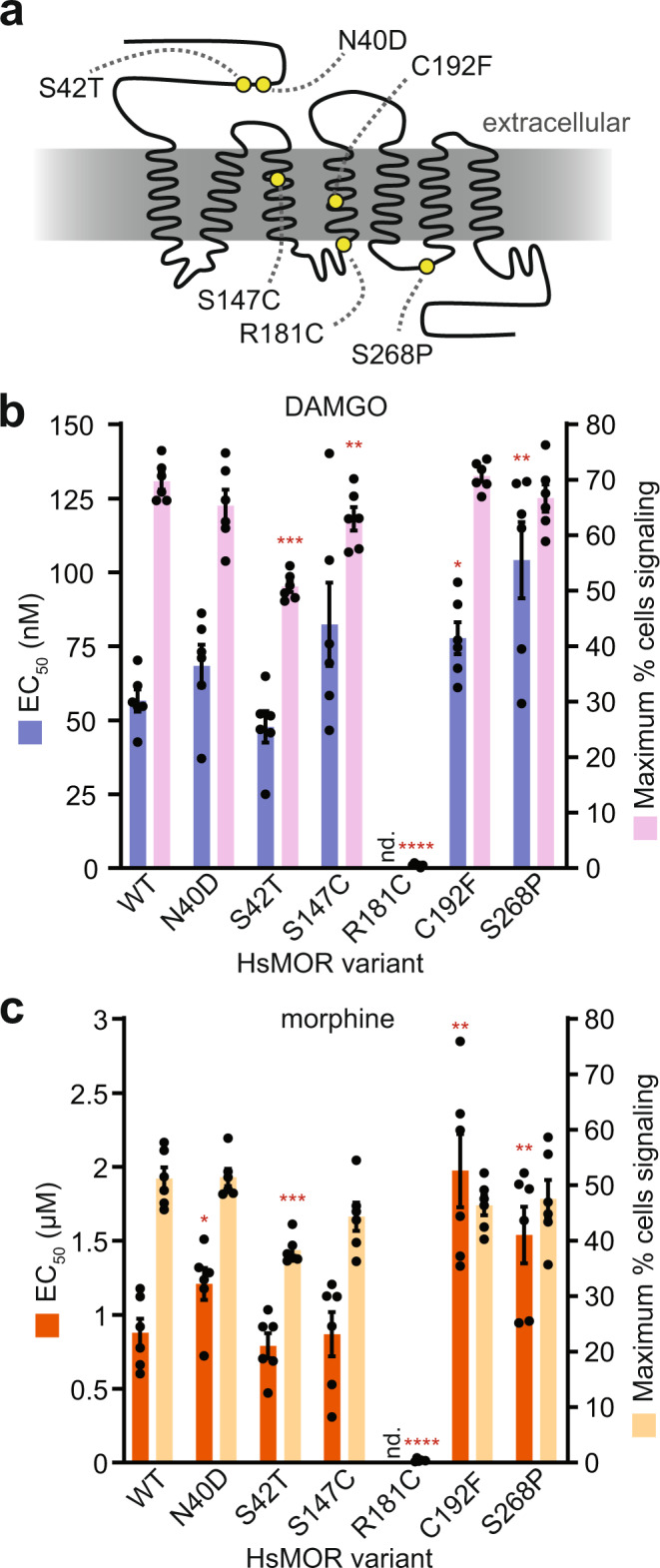
The effects of HsMOR missense mutations on opioid biosensor activity. **a** HsMOR snake plot with previously identified missense mutations. Biosensors based on these mutants were assayed for DAMGO response (**b**), or morphine response (**c**), by flow cytometry. Paired one-way ANOVAs of DAMGO *pEC*_*50*_s and maximum percentage of cells signaling: *n* = 6 biologically independent experiments, >2956 cells/condition/replicate; *P* = 0.0002 and *P* < 0.0001 respectively; Dunnett’s tests against WT shown. Paired one-way ANOVAs of morphine *pEC*_*50*_s and maximum percentage of cells signaling: *n* = 6 biologically independent experiments, >4761 cells/condition/replicate; *P* = 0.0033 and *P* < 0.0001 respectively; Dunnett’s tests against WT shown. Data presented as mean +/− SEM. *, *P* < 0.05; **, *P* < 0.01; ***, *P* < 0.001; ****, *P* < 0.0001. Source data are provided as a Source Data file.

Our biosensor platform may enable convenient characterization of rare opioid receptor alleles. Introduction of receptor variants should allow measurement of altered receptor sensitivities and signaling strength, potentially predicting clinically relevant changes in responses to analgesics. To probe our platform’s ability to detect these changes we tested HsMOR variants, including those with clinical relevance^50,51^, that had previously been characterized in mammalian cell culture experiments (Fig. 4a).

Variant HsMORs were introduced into the biosensor background and response to DAMGO and morphine was measured (Fig. 4b, c and Supplementary Fig. 3C, D). In agreement with previous work, the signal transduction-defective HsMOR(R181C) mutant was unable to respond to either DAMGO or morphine^50–52^. While dramatic defects were clearly detected, alleles associated with subtle defects were also explored. Previous descriptions of the relatively common (8–16% frequency) HsMOR(N40D) allele are more ambiguous, alternately describing no effect on agonist affinities or decreased β-endorphin affinity, while decreased analgesic response to morphine has also been reported^50,53^. In our biosensor, the N40D variant did not differ in DAMGO response though it displayed a decrease in morphine sensitivity (EC_50_ + 38%), consistent with the reported decrease in morphine-based analgesia. Another variant, S268P, has a disrupted phosphorylation site and has been associated with reduced G protein coupling and reduced internalization and desensitization^53^. A HsMOR(S268P)-based biosensor displayed decreased sensitivity to DAMGO (EC_50_ + 84%) and morphine (EC_50_ + 75%), consistent with diminished G protein coupling and raising the possibility of native yeast kinases acting on exogenous GPCRs.

Ravindranathan et al. characterized other HsMOR variants that resulted in mild decreases (S42T, C192F) or an increase (S147C) in sensitivity to DAMGO and morphine^52^. Correspondingly, a HsMOR(S42T)-based biosensor displayed decreased signaling populations with both agonists, and a HsMOR(C192F)-based biosensor had significantly lower sensitivities to DAMGO (EC_50_ + 37%) and morphine (EC_50_ + 125%). However, HsMOR(S147C) did not show improved sensitivity, instead resulting in a mild 10% decrease in the DAMGO-induced signaling population. Thus, the HsMOR biosensor provides a powerful platform to screen variants for changes in activity, which could inform how patients will respond to opioid-based analgesics.

### Exploring the functional significance of HsMOR terminal domains

**Fig. 5:**
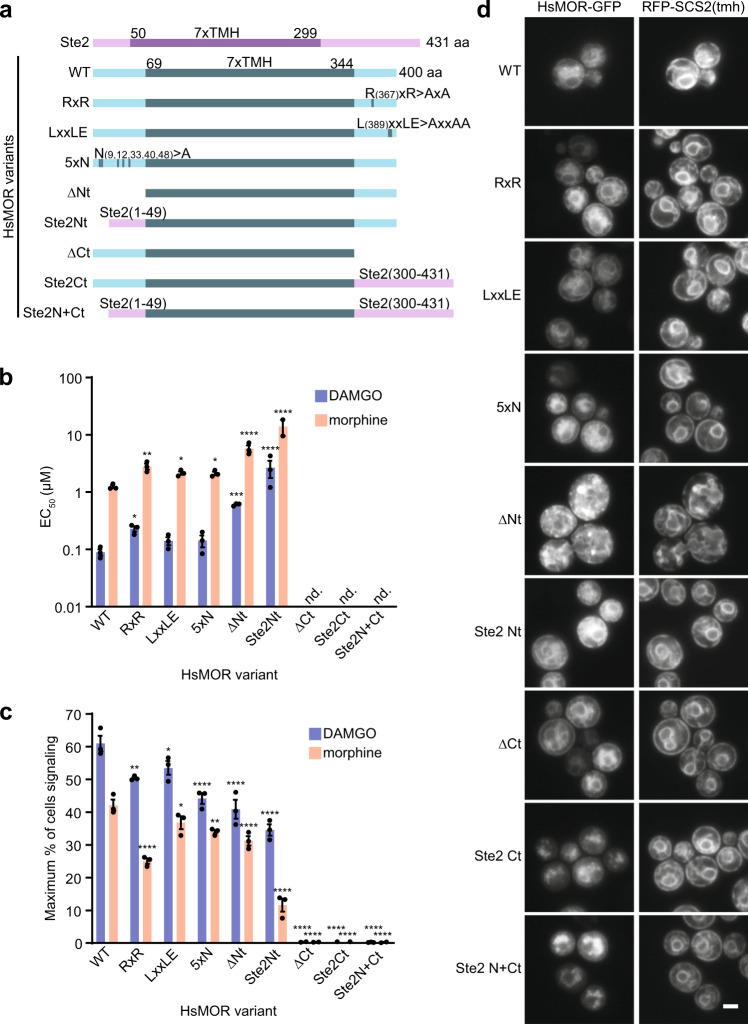
The functional requirements of HsMOR N- and C-terminal domains on biosensor activity. **a** Domains of Ste2, HsMOR, and HsMOR variants with the highly conserved seven transmembrane helix (7xTMH) domains indicated. **b** DAMGO and morphine sensitivity of biosensors based on the indicated HsMOR variants. Unpaired one-way ANOVA of *pEC*_*50*_s: *n* = 3 biologically independent experiments, >7464 cells/strain/replicate; *P* < 0.0001 for both agonists; Dunnett’s tests against WT shown. **c** The effect of N- and C-terminal HsMOR mutations on the population of signaling biosensor cells. Unpaired one-way ANOVA: *n* = 3 biologically independent experiments, >7464 cells/strain/replicate; *P* < 0.0001 for both agonists; Dunnett’s tests against WT shown. **d** Imaging of C-terminally GFP-tagged HsMOR variants in a cholesterol-producing background with ER marker RFP-SCS2(tmh). *n* = 3 biologically independent experiments. Data presented as mean +/− SEM. Scale bar is 2 μm. *, *P* < 0.05; **, *P* < 0.01; ***, *P* < 0.001; ****, *P* < 0.0001. Source data are provided as a Source Data file.

We further applied our platform to explore how additional HsMOR structural variants affect receptor activity and localization in yeast. Opioid receptor terminal domains are moderately conserved, often containing trafficking motifs, glycosylation sites, and phosphorylation sites, collectively contributing to folding, localization, and modification of activity^32,54^. We first made variants lacking putative trafficking motifs R_367_xR and L_389_xxLE, or all five putative N-linked glycosylation sites (Fig. 5a). RxR motifs can bind the coatomer protein I (COPI) complex and have been shown to mediate delta opioid receptor ER/Golgi retention^55^, while LxxLE can be recognized by COPII, facilitating ER exit^56^. N-glycosylation aids in protein quality control and contributes to DOR and KOR folding, stability, and trafficking^57–59^. In response to DAMGO and morphine, biosensors based on all variants displayed subtle decreases in sensitivity (1.6–2.6-fold), suggesting these regions do not greatly contribute to folding or trafficking of HsMOR in yeast (Fig. 5b and Supplementary Fig. 3E, F). Consistently, isoform 2 of HsMOR, which contains a LENLEAETAPLP > VRSL C-terminal substitution and therefore lacks the LxxLE motif, has a similar signaling profile to isoform 1 (Fig. 2b). However, removal of the RxR motif and the N-glycosylation sites did decrease the percent of cells signaling by up to 40 and 28% respectively, highlighting their contribution to achieving optimal activity (Fig. 5c). In line with the overall mild defects, GFP-tagged variants displayed wild type localization (Fig. 5d).

Next, we tested complete removal of the HsMOR N- and C-terminal domains as well as substitution of these domains with those of the endogenous GPCR Ste2, as a small Ste2 N-terminal swap previously improved exogenous GPCR activity^6^. N-terminal deletion decreased DAMGO and morphine sensitivity by 6.8- and 4.6-fold respectively, in line with a previous report of a similar deletion causing a 3.3-fold drop in DAMGO affinity in HEK 293 cells^60^ (Fig. 5b). Thus, the moderate functional contribution of the N-terminus appears conserved. In contrast with previous Ste2 swaps, complete substitution of the HsMOR N-terminus with that of Ste2 also decreased receptor function, reducing HsMOR DAMGO sensitivity 30-fold and decreasing the morphine signaling population by 72% (Fig. 5b, c). However, unlike the N-terminal deletion, which displayed aberrant localization to ER-associated puncta, the N-terminal substitution displayed a wild type localization (Fig. 5d). This suggests the Ste2 N-terminus is sufficient for maintaining localization and that localization poorly correlates with function.

C-terminal domain deletions or Ste2 substitutions also displayed a disconnect between localization and function as they showed no activity while maintaining nearly wild type localization, though with increased vacuolar pools (Fig. 5b–d). The failure of the C-terminal mutants to signal was unexpected as a similar C-terminal deletion displayed only a small reduction in DAMGO sensitivity when expressed in CHO cells^61^. While this may indicate more stringent requirements for activity in yeast, the C-terminal deletions used here disrupt the short cytosolic helix (helix 8) next to the transmembrane domain that, while not involved directly in G protein binding or signal transduction, may contribute to the functional conformation of the receptor^62,63^. Taken together our results show our biosensors can be used to assess how domains and motifs contribute to function, and highlight the difficulty in linking activity to localization.

### Modifying membrane sterols alters HsMOR biosensor function

Cholesterol biosynthetic intermediates are typically present in plasma membranes at low concentrations, and accumulations are linked to developmental and neurological defects^64^. Still, relative proportions of cholesterol and its biosynthetic intermediates can vary based on tissue^65^. It remains unclear to what extent these intermediates can fulfill the roles of cholesterol in promoting GPCR activity. Profiles of sterol intermediates may exist that further promote GPCR signaling in yeast without cholesterol-associated growth and transformation defects^22^.

**Fig. 6:**
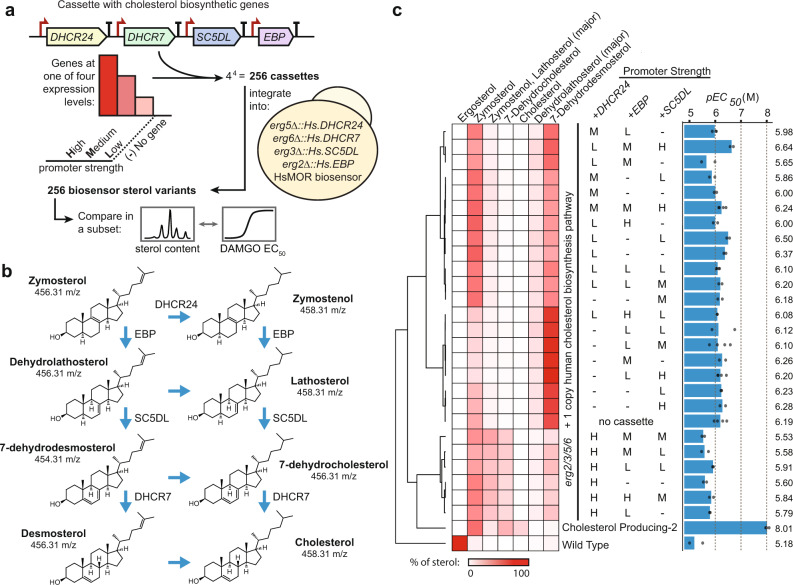
The effect of membrane sterol composition on opioid biosensor signaling efficiency. **a** Construction of an array of putative biosensor strains with systematically varied promoter strength for the cholesterol biosynthetic genes downstream of zymosterol. Cassettes containing these genes under different promoters were integrated into an HsMOR biosensor background with an additional copy of these enzymes in place of the final ergosterol biosynthetic genes (*ERG2/3/5/6*). **b** Cholesterol biosynthesis intermediates after zymosterol. **c** Sterol analysis and full dose responses with DAMGO of 39% of biosensors from the collapsed screen. 10,000 cells/strain/replicate, *n* = 2, 3 or 4. Percentages of total sterol content for the intermediates were determined and mean *pEC*_*50*_s were calculated. Source data are provided as a Source Data file.

To search for sterol profiles that could improve HsMOR-based biosensor performance, we attempted to humanize the cholesterol biosynthetic pathway by introducing genes *DHCR24*, *EBP*, *SC5DL,* and *DHCR7* in a reconstructed *erg2/3/5/6∆* biosensor background using another type of GFP, ZsGreen1, as the reporter (Fig. 1d). Initially, the human genes were introduced at the ergosterol biosynthesis gene loci, and driven by the native yeast promoters (*erg5∆::Hs.DHCR24*, *erg6∆::Hs.DHCR7*, *erg3∆::Hs.SC5DL*, *erg2∆::Hs.EBP*). GC-MS analysis of sterols showed this humanized strain generated the intermediates zymosterol, dehydrolathosterol, and 7-dehydrodesmosterol, while the products of DHCR7 and DHCR24 activity failed to accumulate (Fig. 6b, c; Supplementary Fig 5).

Next we integrated in the yeast genome cassettes containing additional copies of the cholesterol biosynthetic genes under high, medium, or low strength yeast promoters to improve expression and generate strains with modified sterol profiles (Fig. 6a). Of the 256 combinations, 249 were successfully constructed and assayed for response to 10 and 1 µM DAMGO, the concentrations roughly required to reach the E_max_ and EC_50_ in the wild type background (Fig. 1f). Responses ranged from 21 to 61% and 8%to 47% of cells signaling at 10 and 1 µM DAMGO respectively (Supplementary Fig 4A). Human DHCR7 was found to be inactive, confirmed by failure of a Wilcoxon signed-rank test (*P* > 0.05) comparing the strong *DHCR7* expression and no gene conditions, and the absence of products from DHCR7 activity in downstream sterol analyses (Supplementary Fig 4C). Therefore, we excluded *DHCR7* from our analysis and selected 39% of strains from this collapsed set for membrane sterol composition analysis (Supplementary Fig 4A). GC-MS analysis revealed that most variation was in 7-dehydrocholesterol, zymosterol, zymostenol, and lathosterol (Fig. 6c). Subsequently, dose responses using the agonist DAMGO were performed in duplicate, and EC_50_ values for each strain were determined (Fig. 6c and Supplementary Fig 6). We also performed these analyses in similarly constructed biosensors with a wild type ergosterol-producing background and an alternative cholesterol-producing (*erg5∆::TDH3pr-Dr.DHCR7 erg6∆::CCW12pr-Dr.DHCR24;* Cholesterol Producing-2) background.

Hierarchical clustering identified trends in the composition of sterol intermediates. In particular, variations in *DHCR24* promoter strength led to the largest changes in sterol composition, with higher promoter strength correlating with decreased HsMOR sensitivity (Fig. 6c). The single copy of *DHCR24* in the base strain proved insufficient to produce zymostenol, lathosterol, and 7-dehydrocholesterol. Accordingly, the presence of these intermediates correlate with higher EC_50_ values. A linear regression analysis on the sterol intermediate percentages and EC_50_s reinforced the relationship between sterol composition and signaling, finding a strong correlation (Supplementary Fig 4D). The cholesterol-producing biosensor strain proved disproportionally more sensitive with an EC_50_ approximately 24 times lower than the most sensitive strain identified from the screen (Fig. 6c).

### Sterol modifications improve human class A GPCR function in yeast

**Fig. 7:**
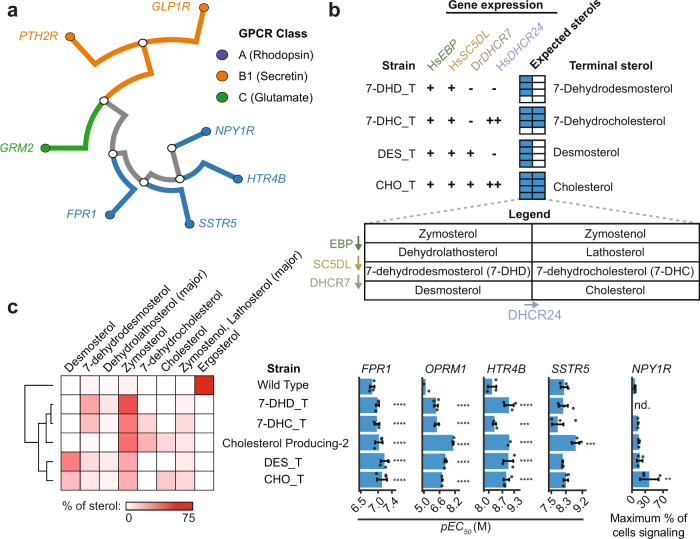
Effect of cholesterol on the activity of different human GPCRs. **a** Phylogenetic relationships of the GPCRs tested. **b** Strains, designated by their terminal sterol, were built to produce all sterols up to the indicated terminal sterols by introducing sterol biosynthetic genes under native ergosterol promoters (+) or strong yeast promoters (++) in a *erg2/3/5/6* biosensor background. (−) indicates no gene is present. The expected sterol space is diagrammed in the expected sterols legend. **c** Sterol profiles of strains with the indicated sterol backgrounds were determined. Biosensors based on expressing HTR4B, FPR1, OPRM1, SSTR5, and NPY1R in all sterol backgrounds were assayed in the presence of their agonists by flow cytometry to construct dose-response curves used to determine pEC_50_s and the maximum percent of cells signaling. Where responses were too weak to determine pEC_50_, maximum percent of cells signaling is reported. Data presented as mean +/− SD. Unpaired one-way ANOVA: *n* = 3 biologically independent experiments except *n* = 2 for HTR4B in Cholesterol Producing-2 and OPRM1 in Wild Type; >10,000 cells/strain/replicate; *P* < 2.2e−16 for *HTR4B*, *FPR1*, *OPRM1*, *SSTR5* assays, *P* = 0.02518 for *NPY1R* assay. Dunnett’s tests against WT shown. *, *P* < 0.05; **, *P* < 0.01; ***, *P* < 0.001; ****, *P* < 0.0001. *OPRM1*, Wild Type, and *OPRM1*, Cholesterol Producing-2 data are replotted from Fig. 6c. Source data are provided as a Source Data file.

To explore how broadly cholesterol improves functional expression of human GPCRs in yeast, we introduced seven different GPCRs into wild type, cholesterol-producing, and sterol intermediate biosensor backgrounds. These receptors belong to three GPCR classes, all can couple with the G_i/o_ chimera, and four of them, *HTR4B*, *GLP1R*, *SSTR5*, and *FPR1*, have been shown to function in yeast^66–69^ (Fig. 7a). Of the resulting putative biosensors, all strains with class A receptors showed response to their cognate agonists at 10 µM and lower, whereas no class B or C receptors signaled in any sterol background (Fig. 7c). Of the receptors reported to be active in yeast, only GLP1R failed to signal, possibly due to the use of different assays.

In order to more generally test these GPCRs’ activities with respect to membrane sterols, as our promoter screen only sampled a subset of intermediate sterols (Fig. 6c), we engineered strains targeting specific terminal sterols (Fig. 7b). This was achieved by selectively expressing a subset of the cholesterol biosynthetic genes in an *erg2/3/5/6* background. Using this panel of strains, we measured dose-response curves for the active receptors (FPR1, SSTR5, HTR4B, and HsMOR) in each sterol background; a dose response could not be measured for *NPY1R* since it only responded to neuropeptide Y concentrations approaching 10 µM. Remarkably, sensitivities of all biosensors were greater in one or more of the modified sterol strains in comparison to the ergosterol-producing wild type strain (Fig. 7c and Supplementary Fig 7). Increases in pEC_50_s with the non-opioid receptors ranged from 2.8-fold (*FPR1*) to 6.5-fold (*HTR4B*) in the best performing strains. EC50 comparisons of the best-performing strains to literature values varied, with *FPR1* 3.5-fold less sensitive than the most-sensitive reported value while *SSTR5* and *HTR4B* were respectively 1.2- and 6.8-fold more sensitive^70–72^. For *NPY1R*, the maximum fraction of cells signaling increased 5.7-fold in the top-performing strain compared to the wild type strain. Thus, the activity of class A GPCRs in yeast can generally be improved by replacing ergosterol with cholesterol and related human sterol intermediates, with different specific sterols preferred by different receptors.

## Discussion

We have shown that engineering yeast to produce cholesterol and other human sterols is an effective strategy for improving vertebrate GPCR activity in yeast, thereby enabling the generation of opioid biosensors with nanomolar sensitivities and expected agonist specificities. This allowed us to evaluate the structural requirements for HsMOR function in yeast and recapitulate many defects associated with clinically relevant missense mutations. Systematic modification of the sterol biosynthetic pathway revealed that while the presence of upstream cholesterol intermediates can improve activity, a cholesterol-producing background is most effective for HsMOR function. The presence of cholesterol and related human sterol intermediates also improved the function of several other GPCRs (FPR1, HTR4B, SSTR5, and NPY1R) indicating that modification of sterols is a general tool for the functional expression of animal GPCRs in yeast.

GPCRs can require cholesterol for normal function or regulation, likely due to both specific GPCR-cholesterol interactions^20^ and non-specific effects such as increased membrane fluidity or the facilitation of lipid subdomains^73^. By comparing GPCR activity in cholesterol- and ergosterol-producing yeast we indirectly assessed the extent to which cholesterol is specifically required for human GPCR activity. Remarkably, cholesterol increased the sensitivity of all tested GPCRs, even though only HsMOR has reported cholesterol-dependence. This suggests that cholesterol often improves human GPCR function beyond a non-specific requirement for sterols in the membrane. Conversely, non-native sterols may actively disrupt function, as Lagane et al. could detect DAMGO binding by HsMOR in yeast lysates only after ergosterol depletion with methyl-β-cyclodextrin^9^. This effect likely contributed to the performance improvements of biosensors producing sterol intermediates. Taken together, the frequency with which GPCRs have evolved to utilize direct interactions with native sterols may be underestimated.

Though many GPCRs benefited from the presence of cholesterol, HsMOR displayed the greatest improvements in sensitivity (Figs. 1 and 7). HsMOR cholesterol dependence was expected as cholesterol is bound in HsMOR crystal structures^62^ and there is evidence that cholesterol directly promotes an active conformation^74^, partitions the receptor into more functional subdomains^75^, and aids in dimerization^76^. Though there is evidence that this receptor could be directly interacting with cholesterol in yeast, the degree to which this is occurring and the mechanism by which this improves activity remains to be resolved. The milder cholesterol dependence of the other receptors likely reflects a more limited potential for cholesterol binding. While cholesterol often improves activity, it has been shown to disrupt the activity of some receptors including the M_2_ muscarinic acetylcholine receptor^77^, type 1 cannabinoid receptors^78^, and rhodopsin^79^. It remains to be determined if the activity of these receptors is similarly disrupted in a cholesterol-producing yeast strain and if rules can be developed to predict which receptors will most benefit from conversion of ergosterol to cholesterol.

Small differences in sterol structure appear to have significant effects on HsMOR signaling. Screening HsMOR-based biosensors producing different sterol intermediates revealed that any combination of cholesterol intermediates increases sensitivity relative to ergosterol, though lathosterol and zymostenol were least beneficial. This was surprising given that in humans enrichment of these intermediates, including zymosterol, lathosterol, and 7-dehydrocholesterol, is disruptive and linked to several diseases^64^. Indeed, one GPCR, *HTR1A*, can be disrupted by increasing 7-dehydrocholesterol levels to mimic Smith–Lemli–Opitz Syndrome^80^. Our sterol intermediate biosensors offer surrogate strategies to screen for other similarly disrupted GPCRs.

Our cholesterol-rich background enabled all opioid receptors to signal, generally with expected agonist specificities, allowing us to establish interspecies conservation of receptor function. The mammalian opioid receptors consistently displayed specificities similar to those of humans, whereas the responses of less related receptors were more variable. Some receptors such as the flying bat MOR, python DOR, or the zebrafish KOR only weakly responded to some of the agonists. In contrast, the bearded dragon KOR had the strongest response to KOR agonists and was also able to respond to the MOR-specific agonist DAMGO. The zebrafish DOR and KOR-based biosensors each showed no response to one of the two type-specific agonists tested, in line with previous work indicating the zebrafish DOR responds more strongly to general agonists than MOR, KOR, or DOR-specific agonists^81^. Indeed, a previous model suggests that there is an increased rate of divergence of mammalian opioid receptors from ancestral receptors relative to those of fish and reptiles, leading to more robust agonist specificities^32^. While our data partially support this model, we find agonist specificities to be widely conserved.

While specificity was well conserved, opioid biosensors were on average 54-fold less sensitive than values previously determined for receptors in more native environments (Fig. 2c). Only the HsDOR met-enkephalin response outperformed reported sensitivities with an EC_50_ of 10 nM, a three-fold improvement. Notably, the drop in sensitivity of MORs in our biosensors was largest and roughly ten times greater than that of DORs, a substantial difference given their close evolutionary and structural relationships (Fig. 2a). Perhaps MORs, which appear to have the highest evolutionary rate^32^, diverged to require additional features of the vertebrate environment for full function. Species of origin was poorly correlated with sensitivity as the origins of the most sensitive mu, delta, and kappa receptors were diverse: mice, humans, and bearded dragons respectively. This indicates that opioid receptor sensitivity may be heavily influenced by sporadic mutations that coincidentally improve performance in yeast.

Thus, there is room to improve opioid biosensor performance, perhaps by further adjusting the biosensor environment or its components. Here, GPCRs were generally codon optimized to improve yeast expression. However, additional tests on a subset of six opioid biosensors, two of each receptor type, found that native genes improve sensitivity by as much as 31-fold for the SNC80 response of PbDOR, and 3.9-fold on average (Supplementary Fig. 1). Though the sensitivity improvement was tempered by a 1.4-fold reduction in percent of cells signaling, using GPCRs with native codons may be beneficial overall. This may be because native genes contain rare codons, which could decrease the rate of translation, potentially promoting the optimal folding of opioid receptors. Other approaches to improve biosensor activity may include strengthening the link to the pheromone response pathway, adding potential chaperones, or performing unbiased screens for yeast deletions that improve activity. Introducing enzymes responsible for post-translational modifications such as palmitoylation^54^ or attempting to adjust yeast membrane thickness^82^ may also be helpful. Alternatively, applying slower biosensor assays that allow greater signal accumulation, such as the 24 h β-galactosidase method used by Olesnicky et al., could improve sensitivity^25^.

Our opioid biosensors and sterol-modified biosensor backgrounds have many applications. The speed and low cost of using our opioid biosensors for screening compounds for receptor type-specific activation should make them an attractive tool to bridge computational docking studies^83^ and more costly screens in human cell lines based on protein complementation^40^ or bioluminescence resonance energy transfer^84^. Currently, our opioid biosensors are unable to measure modes of signaling beyond G protein activation, such as β-arrestin recruitment, which is thought to cause many of the side effects of opioids. This makes the biosensors less useful for drug discovery efforts which are focused on identifying compounds that display biased agonism towards G protein activation. However, our biosensors are compatible with the PRESTO-Tango^85^ system for detecting GPCR-β-arrestin interactions, which would allow future biosensors to detect biased agonism. By increasing throughput of production assays from hundreds to thousands, these biosensors will also aid in the ongoing development of opiate production strains^86^. Furthermore, it may be possible to adapt the opioid biosensors for opioid-detecting field tests. Colorimetric assays based on yeast biosensors have been reported previously^87^, and in principle, our biosensors could be used to test a sample for the degree of opioid activity independent of identifying the compounds present. This may enable testing kits that could be used to assess the amount of a sample likely to cause an overdose. Other direct biotechnology applications might leverage library-based approaches to discover drugs and/or GPCR variants, with the potential for particularly high-throughput/low-cost variant screening. Beyond opioid biosensors, our sterol-modified platform should enable the expression of many other human GPCRs in yeast, generating an array of new biosensors and tools for the deorphanization of GPCRs.

## Methods

### Strains and plasmids

Strains and plasmids are listed in Supplementary Data 1 and 2. Strains were derived from BY4741^88^ using CRISPR-Cas9 as follows. A Cas9 (CEN6 *URA3*) vector was constructed using components of the Yeast Toolkit^89^, with *pPGK1*-Cas9-*tENO2* and up to four sgRNAs expressed from a tRNA^Phe^ promoter with a 5′ HDV ribozyme site and a *SNR52* terminator. Alternatively, the MyLO CRISPR-Cas9 vector system was used^90^. Strains were constructed by transforming yeast with a Cas9 vector, unique protospacers guiding Cas9, and a double stranded repair template introducing deletions or modifications. Deletions and modifications were confirmed by colony PCR and sequencing respectively. All protospacers and repair template sequences are listed in Supplementary Data 3. Yeast was transformed using either the Zymo Research EZ Yeast Transformation II Kit (cat. T2001) or a modified Gietz protocol^91^.

Plasmids were constructed using Golden Gate assembly of components from the Yeast Toolkit^89^ and elsewhere. Opioid receptors were all expressed from the same 2 µ HIS3 backbone assembly (ConLS’-*CCW12p*-GPCR-*SSA1t*-ConRE’-*HIS3*-*2μ*-*KanR*-ColE1), while *FPR1* was on a similar vector with a *TDH1* terminator and other GPCRs were expressed from a ConLS’-*CCW12p*-GPCR-*SSA1t*-ConRE’-URA3-2μ-KanR-p15a backbone. GPCRs were ordered as either gblocks from IDT or clonal genes from Twist Biosciences. GPCR sequences are listed in Supplementary Data 4 and were yeast codon optimized unless specified as non-Codon Optimized (nCO).

### Media and GPCR effectors used

For all signaling assays with human receptors, overnight cultures were back-diluted into selective media at pH 7.1. Media was buffered with 100 mM MOPS or Tris-Cl and the pH was adjusted with NaOH or HCl, respectively. All GPCR effectors used are listed in Supplementary Data 7.

### Sterol extraction

Yeast strains were grown to either mid-log (8hrs) or saturation (48 h) from single colonies. Since the growth rates of these strains were different, wet weights were adjusted to 50 and 150 mg for the 8 and 48 h timepoints respectively. These were then suspended in glass tubes containing 3 ml of 10% w/v methanolic KOH. The tubes were flushed with nitrogen gas and capped before incubating at 70 °C for 90 min. Samples were cooled to room temperature before 1 ml of water and 2 ml of n-hexane were added and vortexed. The hexane phase was transferred to glass vials and the extraction process was repeated. Combined extracts were dried under nitrogen and derivatized by adding 50 µl N,O-Bis(trimethylsilyl)trifluoroacetamide:Trimethylchlorosilane (BSTFA, 1% TMCS) and incubating at 60 °C for 30 min. Derivatized samples were dried under nitrogen or by vacuum centrifugation for ~30 min, and finally suspended in ethyl acetate for GC-MS analysis.

### GC-MS analysis of sterols

Derivatized sterol extracts and standards were analyzed on an Agilent Technologies 5977 GC/MSD equipped with a Agilent J&W DB-1MS UI capillary column with 45 m in length, 0.25 mm inner diameter, and 0.25 µm phase thickness (phase- 100% dimethylpolysiloxane). Sterols from 1 µl injections were separated using an initial oven temperature of 40 °C for 1 min followed by a 20 °C/min ramp to 320 °C, which was held for 12 min (constant helium flow of 1 ml/min). The mass spectrometer source and transfer line temperatures were set at 260 and 280 °C, respectively and the GC inlet was operated in splitless mode. Mass spectral data were analyzed using MassHunter Workstation Software version B.06.00 (Agilent). Parent and fragment ion counts were extracted at 129.3, 454.3, 456.3, 458.3, and 468.3 m/z using a window of +/− 0.5 m/z for analysis. Extracted Ion Chromatograms (EICs) were aligned, then individual sterols quantified as baseline-corrected peak areas across appropriate retention time windows for the following ions: 454.3, 7-dehydrodesmosterol; 456.3, 7-dehydrocholesterol, zymosterol, 7-dehydrolathosterol; 458.3, cholesterol, zymostenol+lathosterol; 468.3, ergosterol. Relative sterol abundances were calculated as the percentage of total ions detected for the set of measured sterols. Ambiguities between 7-dehydrocholesterol and desmosterol were resolved by examination of the 129/456 fragment ion ratio, and assignments were confirmed using purified standards as shown in Supplementary Fig 5.

### Plate reader signaling assay

Yeast was grown overnight in synthetic selective media and back-diluted 1:10 into media, with agonists as indicated, in Falcon 96 well microtiter plates to 100 µL final volumes. Cells were shaken at 30 °C for either 3 h (alpha mating factor tests) or 8 h (DAMGO tests) prior to measurement on a CLARIOstar plate reader with software version 5.21.R4 (BMG Labtech). Values for OD600 and green fluorescence (excitation 469 nm ± 13 nm, emission 508 nm ± 15 nm) or red fluorescence (excitation 527 nm ± 27 nm, emission 622 nm ± 30 nm) were collected for each sample.

### Flow cytometer signaling assay

Overnight cultures grown in synthetic selective media were back-diluted 1:10 into fresh media containing the agonist being tested to a final volume of 100 µL in a Falcon 96 well microtiter plate. Agonists were typically tested with at least seven concentrations in a five-fold dilution series except in Fig. 1f where ten concentrations in a three-fold dilution series were used. Cells were shaken at 300 rpm for 8 h (or 6 h for alpha mating factor tests) prior to measurement on an BD Accuri C6 flow cytometer with CFlow Plus 1.0.227.4. Either 10,000 events, or those within 15 µL of the culture, were recorded. For alpha mating factor response measurements the mean green fluorescence of the complete, ungated population was determined and used to calculate fold induction of fluorescence. Otherwise, within an experiment the biosensor that was brightest in its inactive state (no agonist) was used to establish an arbitrary green fluorescence intensity threshold such that 0.1–1% of cells were brighter than the threshold. This threshold was propagated to all conditions within the experiment and the percentage of the cells within each measurement that exceeded the threshold were recorded as the percentage of cells signaling. The percentage of cells signaling was exported to Excel 16.0.14326.20908 (Microsoft) and processed before constructing 4 parameter dose-response curves within Prism 8.4.3 (GraphPad) to determine EC50s, IC50s, and the maximum percentage of cells signaling within a biosensor-agonist condition.

Alternatively, for Figs. 6 and 7, overnight cultures were back-diluted to an OD600 of approximately 0.2. The agonist was added upon dilution and cells were grown for 8 h in 96-well deep well plates at a volume of 500 µl at 30 °C with shaking at 1000 rpm. 10,000 singlet cells of each sample were analyzed using a SP6800 Spectral Analyzer (Sony).

### Microscopy

Log phase yeast grown in synthetic selective media were mounted on slides and imaged using a DMi6000B microscope (Leica Microsystems) with an HCX PL APO 63× oil objective, an Orca R2 CCD camera (Hamamatsu), and Volocity 5.5.1 software (PerkinElmer). Images were processed using FiJi 1.51 23^92^ and Photoshop 2015.0.0 (Adobe), and assembled in Illustrator 19.0.0 (Adobe).

### Statistics and reproducibility

No statistical method was used to predetermine sample size. Sample sizes used were in line with generally accepted standards, such as those in Lú Chau et al.^93^. No data were excluded from the analyses. The experiments were not randomized. The investigators were not blinded to allocation during experiments and outcome assessment.

### Reporting summary

Further information on research design is available in the Nature Research Reporting Summary linked to this article.

## Supplementary information

Supplementary Information

Reporting Summary

Supplementary Data 1 - Yeast Strains Used

Supplementary Data 2 - Plasmids used

Supplementary Data 3 - Sequences used for transformations

Supplementary Data 4 - DNA sequences used

Supplementary Data 5 - Literature GPCR sensitivities

Supplementary Data 6 - Additional metrics of biosensor activity

Supplementary Data 7 - Sources of GPCR effectors used

Description of Additional Supplementary Files

## Data Availability

Source data are provided with this paper. Further raw acquisition files and extended data sets are available from the corresponding authors on request. Source data are provided with this paper.

## Source data

Source Data

## References

[1] Pierce KL, Premont RT, Lefkowitz RJ (2002). Seven-transmembrane receptors. Nat. Rev. Mol. Cell Biol..

[2] Hauser AS, Attwood MM, Rask-Andersen M, Schiöth HB, Gloriam DE (2017). Trends in GPCR drug discovery: New agents, targets and indications. Nat. Rev. Drug Discov..

[3] Elion EA (2000). Pheromone response, mating, and cell biology. Curr. Opin. Microbiol..

[4] Alvaro CG, Thorner J (2016). Heterotrimeric G protein-coupled receptor signaling in yeast mating pheromone response. J. Biol. Chem..

[5] Brown AJ (2000). Functional coupling of mammalian receptors to the yeast mating pathway using novel yeast/mammalian G protein a-subunit chimeras. Yeast.

[6] King K, Dohlman HG, Thorner J, Caron MG, Lefkowitz RJ (1990). Control of yeast mating signal transduction by a mammalian β-adrenergic receptor and Gs α subunit. Science.

[7] Lengger B, Jensen MK (2020). Engineering G protein-coupled receptor signalling in yeast for biotechnological and medical purposes. FEMS Yeast Res..

[8] Sarramegna V, Talmont F, Demange P, Milon A (2003). Heterologous expression of G-protein-coupled receptors: Comparison of expression systems from the standpoint of large-scale production and purification. Cell. Mol. Life Sci. CMLS.

[9] Lagane B (2000). Role of sterols in modulating the human μ-opioid receptor function in *Saccharomyces cerevisiae*. J. Biol. Chem..

[10] O’Malley MA (2009). Progress toward heterologous expression of active G-protein-coupled receptors in *Saccharomyces cerevisiae*: Linking cellular stress response with translocation and trafficking. Protein Sci..

[11] Butz JA, Niebauer RT, Robinson AS (2003). Co-expression of molecular chaperones does not improve the heterologous expression of mammalian G-protein coupled receptor expression in yeast. Biotechnol. Bioeng..

[12] Yoo JI, O’Malley MA (2018). Tuning vector stability and integration frequency elevates functional GPCR production and homogeneity in *Saccharomyces cerevisiae*. ACS Synth. Biol..

[13] Schütz M (2016). Directed evolution of G protein-coupled receptors in yeast for higher functional production in eukaryotic expression hosts. Sci. Rep..

[14] Ritter SL, Hall RA (2009). Fine-tuning of GPCR activity by receptor-interacting proteins. Nat. Rev. Mol. Cell Biol..

[15] Chan HCS (2020). Enhancing the signaling of GPCRs via orthosteric ions. ACS Cent. Sci..

[16] Opekarová M, Tanner W (2003). Specific lipid requirements of membrane proteins—a putative bottleneck in heterologous expression. Biochim. Biophys. Acta BBA - Biomembr..

[17] Paila, Y. D. & Chattopadhyay, A. *Cholesterol Binding and Cholesterol Transport Proteins* (ed. Harris, J. R.) Vol. 51, 439–466 (Springer Netherlands, 2010).

[18] Genheden S, Essex JW, Lee AG (2017). G protein coupled receptor interactions with cholesterol deep in the membrane. Biochim. Biophys. Acta BBA - Biomembr..

[19] Guixà-González R (2017). Membrane cholesterol access into a G-protein-coupled receptor. Nat. Commun..

[20] Taghon GJ, Rowe JB, Kapolka NJ, Isom DG (2021). Predictable cholesterol binding sites in GPCRs lack consensus motifs. Structure.

[21] Morioka S (2013). Effect of sterol composition on the activity of the yeast G-protein-coupled receptor Ste2. Appl. Microbiol. Biotechnol..

[22] Souza CM (2011). A stable yeast strain efficiently producing cholesterol instead of ergosterol is functional for tryptophan uptake, but not weak organic acid resistance. Metab. Eng..

[23] Waldhoer M, Bartlett SE, Whistler JL (2004). Opioid receptors. Annu. Rev. Biochem..

[24] Skolnick P (2018). The opioid epidemic: Crisis and solutions. Annu. Rev. Pharmacol. Toxicol..

[25] Olesnicky NS, Brown AJ, Dowell SJ, Casselton LA (1999). A constitutively active G-protein-coupled receptor causes mating self-compatibility in the mushroom Coprinus. EMBO J..

[26] Erdman S, Lin L, Malczynski M, Snyder M (1998). Pheromone-regulated genes required for yeast mating differentiation. J. Cell Biol..

[27] Heiman MG, Walter P (2000). Prm1p, a pheromone-regulated multispanning membrane protein, facilitates plasma membrane fusion during yeast mating. J. Cell Biol..

[28] Erickson JR (1998). Edg-2/Vzg-1 couples to the yeast pheromone response pathway selectively in response to lysophosphatidic acid. J. Biol. Chem..

[29] Casey JR, Grinstein S, Orlowski J (2010). Sensors and regulators of intracellular pH. Nat. Rev. Mol. Cell Biol..

[30] Harris I, Jones EW, Aldred CN (1934). The pH and lactic acid content of the cerebrospinal fluid. J. Neurol. Neurosurg. Psychiatry.

[31] Edgar RC (2004). MUSCLE: Multiple sequence alignment with high accuracy and high throughput. Nucleic Acids Res..

[32] Stevens CW (2004). The evolution of vertebrate opioid receptors. Front. Biosci..

[33] Raynor K (1994). Pharmacological characterization of the cloned kappa-, delta-, and mu-opioid receptors. Mol. Pharmacol..

[34] Toll L (1998). Standard binding and functional assays related to medications development division testing for potential cocaine and opiate narcotic treatment medications. NIDA Res. Monogr..

[35] Nickolls SA, Waterfield A, Williams RE, Kinloch RA (2011). Understanding the effect of different assay formats on agonist parameters: A study using the µ-opioid receptor. J. Biomol. Screen..

[36] Kreil G (1989). Deltorphin, a novel amphibian skin peptide with high selectivity and affinity for delta opioid receptors. Eur. J. Pharmacol..

[37] Bilsky J (1995). SNC 80, a selective, nonpeptidic, and systemically active opioid delta agonist. J. Pharmacol. Exp. Ther..

[38] Camilleri M (2008). Novel pharmacology: Asimadoline, a κ-opioid agonist, and visceral sensation. Neurogastroenterol. Motil..

[39] Yasuda K (1993). Cloning and functional comparison of kappa and delta opioid receptors from mouse brain. Proc. Natl Acad. Sci. USA.

[40] Burford NT (2014). Identification of selective agonists and positive allosteric modulators for µ- and δ-opioid receptors from a single high-throughput screen. J. Biomol. Screen..

[41] Tonini M (1998). Endomorphin-1 and endomorphin-2 activate µ-opioid receptors in myenteric neurons of the guinea-pig small intestine: *Naunyn*. Schmiedebergs Arch. Pharmacol..

[42] LaVigne J, Keresztes A, Chiem D, Streicher JM (2020). The endomorphin-1/2 and dynorphin-B peptides display biased agonism at the mu opioid receptor. Pharmacol. Rep..

[43] Broad J (2016). Human native kappa opioid receptor functions not predicted by recombinant receptors: Implications for drug design. Sci. Rep..

[44] Malatynska E (1996). Human delta opioid receptor: Functional studies on stably transfected Chinese hamster ovary cells after acute and chronic treatment with the selective nonpeptidic agonist SNC-80. J. Pharmacol. Exp. Ther..

[45] Knapp RJ (1996). Structure-activity relationships for SNC80 and related compounds at cloned human delta and mu opioid receptors. J. Pharmacol. Exp. Ther..

[46] Quitterer U, Pohl A, Langer A, Koller S, AbdAlla S (2011). A cleavable signal peptide enhances cell surface delivery and heterodimerization of Cerulean-tagged angiotensin II AT1 and bradykinin B2 receptor. Biochem. Biophys. Res. Commun..

[47] Martoglio B, Dobberstein B (1998). Signal sequences: More than just greasy peptides. Trends Cell Biol..

[48] Rutz, C., Klein, W. & Schülein, R. *Progress in Molecular Biology and Translational Science* Vol. 132, 267–287 (Elsevier, 2015). 10.1016/bs.pmbts.2015.03.003 26055063

[49] Fitzgerald I, Glick BS (2014). Secretion of a foreign protein from budding yeasts is enhanced by cotranslational translocation and by suppression of vacuolar targeting. Microb. Cell Factories.

[50] Mayer P, Höllt V (2001). Allelic and somatic variations in the endogenous opioid system of humans. Pharmacol. Ther..

[51] Olsen T, Rasmussen A, Kringen MK, Molden E (2019). A girl of early school-age with no response to opioids during general anaesthesia. Tidsskr. Den. Nor. Legeforening.

[52] Ravindranathan A (2009). Functional characterization of human variants of the mu-opioid receptor gene. Proc. Natl Acad. Sci. USA.

[53] Lötsch J, Geisslinger G (2005). Are μ-opioid receptor polymorphisms important for clinical opioid therapy?. Trends Mol. Med..

[54] Lemos Duarte M, Devi LA (2020). Post-translational modifications of opioid receptors. Trends Neurosci..

[55] Shiwarski DJ, Crilly SE, Dates A, Puthenveedu MA (2019). Dual RXR motifs regulate nerve growth factor-mediated intracellular retention of the delta opioid receptor. Mol. Biol. Cell.

[56] Geva Y, Schuldiner M (2014). The back and forth of cargo exit from the endoplasmic reticulum. Curr. Biol..

[57] Li J-G, Chen C, Liu-Chen L-Y (2007). *N*-Glycosylation of the human κ opioid receptor enhances its stability but slows its trafficking along the biosynthesis pathway. Biochemistry.

[58] Lackman JJ, Markkanen PMH, Hogue M, Bouvier M, Petäjä-Repo UE (2014). N-Glycan-dependent and -independent quality control of human δ opioid receptor N-terminal variants. J. Biol. Chem..

[59] Markkanen PMH, Petäjä-Repo UE (2008). N-Glycan-mediated quality control in the endoplasmic reticulum is required for the expression of correctly folded δ-opioid receptors at the cell surface. J. Biol. Chem..

[60] Chaturvedi K, Shahrestanifar M, Howells RD (2000). mu Opioid receptor: Role for the amino terminus as a determinant of ligand binding affinity. Mol. Brain Res..

[61] Deng HB (2000). Role for the C-terminus in agonist-induced μ opioid receptor phosphorylation and desensitization. Biochemistry.

[62] Manglik A (2012). Crystal structure of the µ-opioid receptor bound to a morphinan antagonist. Nature.

[63] Feng G-J (2003). Selective interactions between Helix VIII of the human μ-opioid receptors and the C terminus of periplakin disrupt G protein activation. J. Biol. Chem..

[64] Platt FM (2014). Disorders of cholesterol metabolism and their unanticipated convergent mechanisms of disease. Annu Rev. Genomics Hum. Genet..

[65] Cook, R. P. *Distribution of Sterols in Organisms and in Tissues. In Cholesterol: Chemistry, Biochemistry, and Pathology* 145–180 (Academic Press, 1958).

[66] Ehrenworth AM, Claiborne T, Peralta-Yahya P (2017). Medium-throughput screen of microbially produced serotonin via a G-protein-coupled receptor-based sensor. Biochemistry.

[67] Weston C, Poyner D, Patel V, Dowell S, Ladds G (2014). Investigating G protein signalling bias at the glucagon‐like peptide‐1 receptor in yeast. Br. J. Pharmacol..

[68] Ishii J (2012). Cell wall trapping of autocrine peptides for human G-protein-coupled receptors on the yeast cell surface. PLoS One.

[69] Klein C (1998). Identification of surrogate agonists for the human FPRL-1 receptor by autocrine selection in yeast. Nat. Biotechnol..

[70] Crocetti L (2013). Synthesis and pharmacological evaluation of new pyridazin-based thioderivatives as formyl peptide receptor (FPR) agonists: FPR mixed agonists. Drug Dev. Res..

[71] Grant M, Alturaihi H, Jaquet P, Collier B, Ujendra U (2008). Cell growth inhibition and functioning of human somatostatin receptor type 2 are modulated by receptor heterodimerization. Mol. Endocrinol..

[72] Pindon A (2002). Differences in signal transduction of two 5-HT4 receptor splice variants: Compound specificity and dual coupling with G␣s- and G␣i/o-proteins. Mol. Pharmacol..

[73] Jafurulla M, Kumar AdityaG, Rao BD, Chattopadhyay A (2019). A critical analysis of molecular mechanisms underlying membrane cholesterol sensitivity of GPCRs. Adv. Exp. Med. Biol..

[74] Gaibelet G (2008). Cholesterol content drives distinct pharmacological behaviours of µ-opioid receptor in different microdomains of the CHO plasma membrane. Mol. Membr. Biol..

[75] Levitt ES, Clark MJ, Jenkins PM, Martens JR, Traynor JR (2009). Differential effect of membrane cholesterol removal on mu- and delta-opioid receptors: A parallel comparison of acute and chronic signaling to adenylyl cyclase. J. Biol. Chem..

[76] Zheng H (2012). Palmitoylation and membrane cholesterol stabilize μ-opioid receptor homodimerization and G protein coupling. BMC Cell Biol..

[77] Michal P, Rudajev V, El-Fakahany EE, Doležal V (2009). Membrane cholesterol content influences binding properties of muscarinic M2 receptors and differentially impacts activation of second messenger pathways. Eur. J. Pharmacol..

[78] Bari M, Paradisi A, Pasquariello N, Maccarrone M (2005). Cholesterol-dependent modulation of type 1 cannabinoid receptors in nerve cells. J. Neurosci. Res..

[79] Albert A, Boeszebattaglia K (2005). The role of cholesterol in rod outer segment membranes. Prog. Lipid Res..

[80] Paila YD, Murty MRVS, Vairamani M, Chattopadhyay A (2008). Signaling by the human serotonin1A receptor is impaired in cellular model of Smith–Lemli–Opitz Syndrome. Biochim. Biophys. Acta BBA - Biomembr..

[81] Rodriguez, R. E. et al. Characterization of ZFOR1, a putative delta-opioid receptor from the teleost zebrafish (Danio rerio). *Neurosci. Lett*. **4** 207–210 (2000). 10.1016/s0304-3940(00)01239-8 10889344

[82] Sharpe HJ, Stevens TJ, Munro S (2010). A comprehensive comparison of transmembrane domains reveals organelle-specific properties. Cell.

[83] Manglik A (2016). Structure-based discovery of opioid analgesics with reduced side effects. Nature.

[84] Ehrlich AT (2019). Biased signaling of the Mu opioid receptor revealed in native neurons. iScience.

[85] Kroeze WK (2015). PRESTO-Tango as an open-source resource for interrogation of the druggable human GPCRome. Nat. Struct. Mol. Biol..

[86] Pyne ME (2020). A yeast platform for high-level synthesis of tetrahydroisoquinoline alkaloids. Nat. Commun..

[87] Ostrov N (2017). A modular yeast biosensor for low-cost point-of-care pathogen detection. Sci. Adv..

[88] Brachmann, C. B. et al. Designer deletion strains derived from Saccharomyces cerevisiae S288C: A useful set of strains and plasmids for PCR‐mediated gene disruption and other applications. **14**, 115–132 (1998). 10.1002/(SICI)1097-0061(19980130)14:2<115::AID-YEA204>3.0.CO;2-2 9483801

[89] Lee ME, DeLoache WC, Cervantes B, Dueber JE (2015). A highly characterized yeast toolkit for modular, multipart assembly. ACS Synth. Biol..

[90] Bean, B. D. M., Whiteway, M. & Martin, V. J. J. The MyLo CRISPR-Cas9 Toolkit: A markerless yeast localization and overexpression CRISPR-Cas9 Toolkit. Preprint at *bioRxiv*10.1101/2021.12.15.472800 (2022). 10.1093/g3journal/jkac154 PMC9339301 35708612

[91] Gietz RD, Schiestl RH (2007). High-efficiency yeast transformation using the LiAc/SS carrier DNA/PEG method. Nat. Protoc..

[92] Schindelin J (2012). Fiji: An open-source platform for biological-image analysis. Nat. Methods.

[93] Lú Chau T, Guillan A, Roca E, Nunez MJ, Lema JM (2001). Population dynamics of a continuous fermentation of recombinant saccharomyces cerevisiae using flow cytometry. Biotechnol. Prog..

