# Supplementary material for: Functional expression of opioid receptors and other human GPCRs in yeast engineered to produce human sterols
Source: Nat Commun. 2022 May 24;13:2882. doi: 10.1038/s41467-022-30570-7 (PMC9130329; doi:10.1038/s41467-022-30570-7)
Supplement: Supplementary file 1 — Supplementary Information [file 41467_2022_30570_MOESM1_ESM.pdf]

Bean *et al.* Supplementary Figures

Supplementary Figure 1 - Additional metrics of biosensor activity

Supplementary Figure 2 - Dose-response curves of putative opioid biosensors against established agonists and antagonists

Supplementary Figure 3 - DAMGO and morphine dose-response curves of biosensors based on introducing HsMOR variants into the cholesterol-producing chassis

Supplementary Figure 4 - Characteristics of cholesterol biosynthesis intermediate screen strains

Supplementary Figure 5 - Consistency of sterol proportions across biological and technical replicates

Supplementary Figure 6 - Dose-response curves of HsMOR in sterol variant strains

Supplementary Figure 7 - Dose-response curves of HsMOR and non-opioid receptors in sterol variant strains

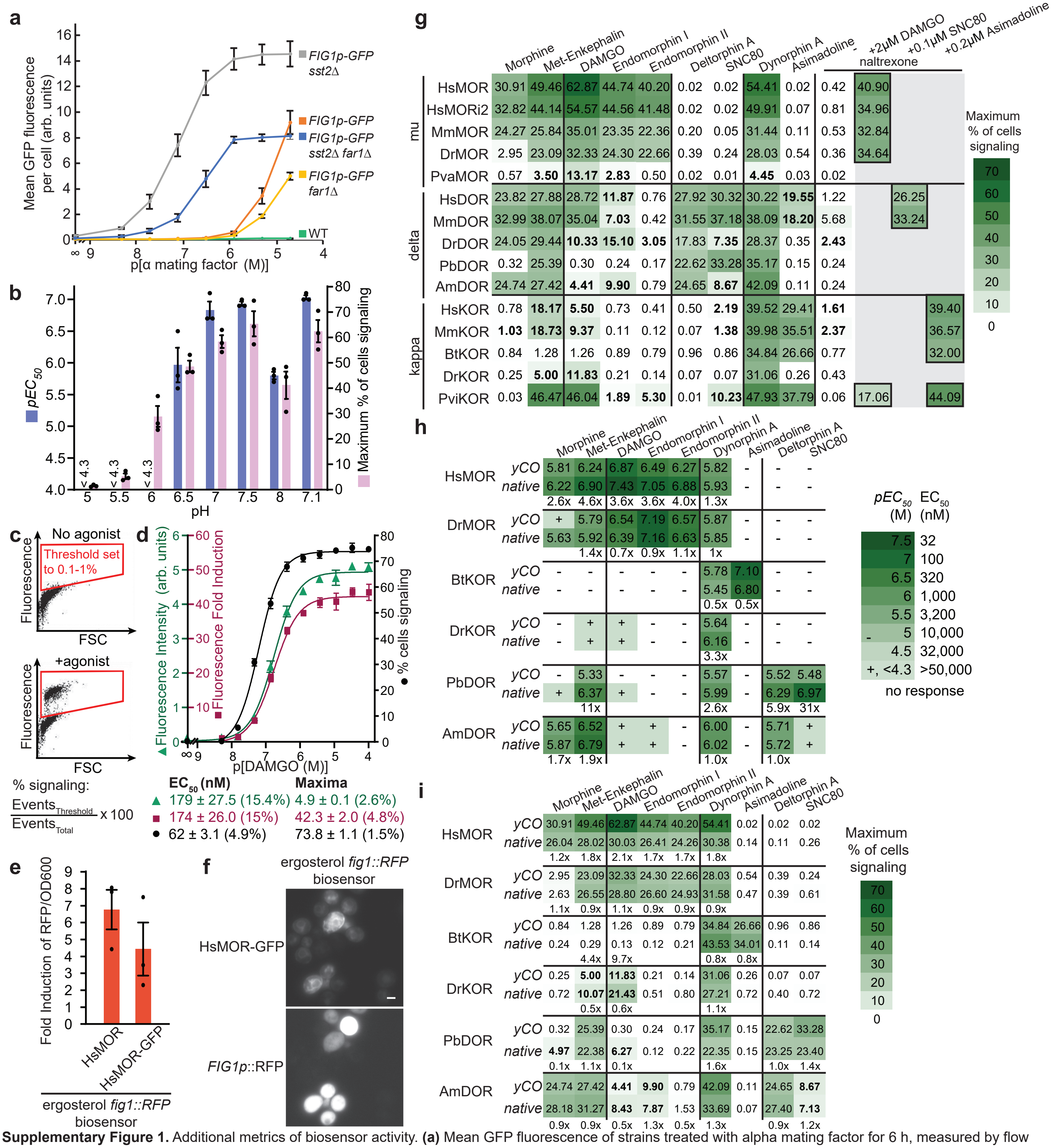

Supplementary Figure 1. Additional metrics of biosensor activity. **(a)** Mean GFP fluorescence of strains treated with alpha mating factor for 6 h, measured by flow cytometry. n=3 biologically independent experiments; 10,000 cells/strain/replicate. **(b)** pH dependence of the HsMOR-based cholesterol-producing biosensor response to DAMGO for 8 h measured by flow cytometry. Media buffered with 100 mM Tris. n=3 biologically independent experiments, >8835 cells/condition/replicate. **(c)** Schematic of how the percent of cells signaling was determined by flow cytometry. A plot of fluorescence against forward scattered light (FSC) for an inactive biosensor was used to set a threshold that 0.1-1% of cells passed, which was then used to determine percent signaling as shown. **(d)** DAMGO dose-response curves for the HsMOR-based cholesterol-producing biosensor generated using three measurement types (green fluorescence, fold induction of fluorescence and percent of cells signaling) demonstrates percent of cells signaling is most sensitive. Data correspond with Figure 1f. n=3 biologically independent experiments; 10000 cells/strain/replicate. **(e)** The effect of GFP tagging the HsMOR C-terminus in an ergosterol biosensor background with a RFP reporter, as measured by a plate reader after 8h of 10 µM DAMGO agonist exposure. n=3 biologically independent experiments. **(f)** Imaging of HsMOR-GFP biosensor after 6 h activation by 10 µM DAMGO. n=3 biologically independent experiments; scale bar is 2 µm. **(g)** Measurement of the maximum percent of biosensor cells signaling in the experiment presented in Figure 3B. n=3 biologically independent experiments, >2073 cells/condition/replicate. **(h)** *pEC50* and **(i)** maximum percent of cells signaling for a subset of opioid biosensors based on yeast codon-optimized receptors compared with receptors expressed from genes with native codon usage. Measurements were taken after 8 h treatment with the indicated agonists, samples were measured by flow cytometry. Experiments run in parallel with that of panel **e**. n=3 biologically independent experiments, >2073 cells/condition/replicate. Fold increase in native receptor EC50 and fold decrease in native receptor signaling populations are indicated. Data presented as mean +/- SEM. Source data are provided as a Source Data file.

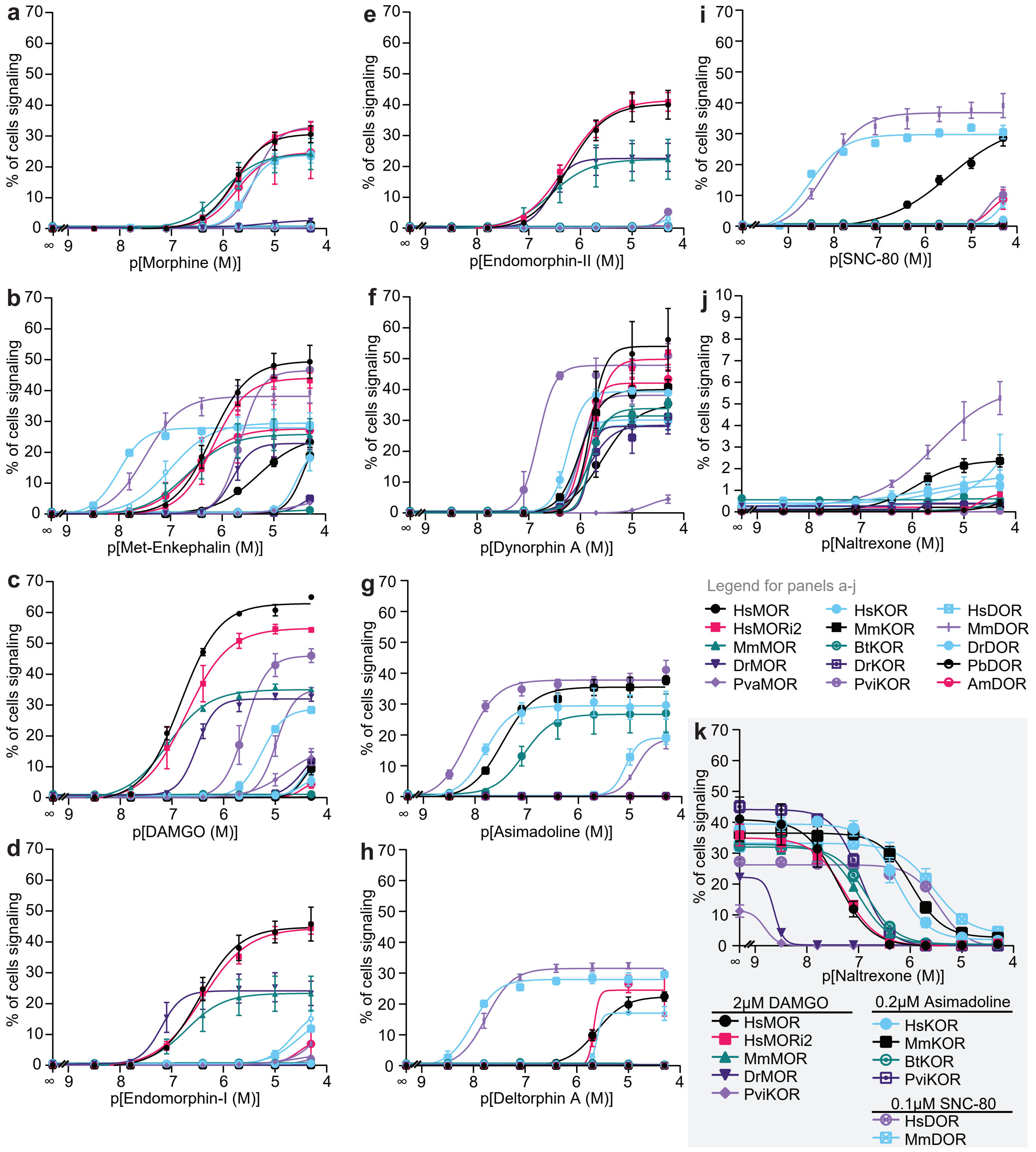

Supplementary Figure 2. Dose-response curves of putative opioid biosensors against established agonists and antagonists. **(a-j)** Biosensor strain response to the indicated agonists were measured and fit using a 4 parameter nonlinear model. Note differing axis scale in j. **(k)** The ability of naltrexone to block activation by the indicated agonists was determined. For **a-k**, n=3 biologically independent experiments; >2073 cells/condition/replicate; data presented as mean +/- SEM. Source data are provided as a Source Data file.

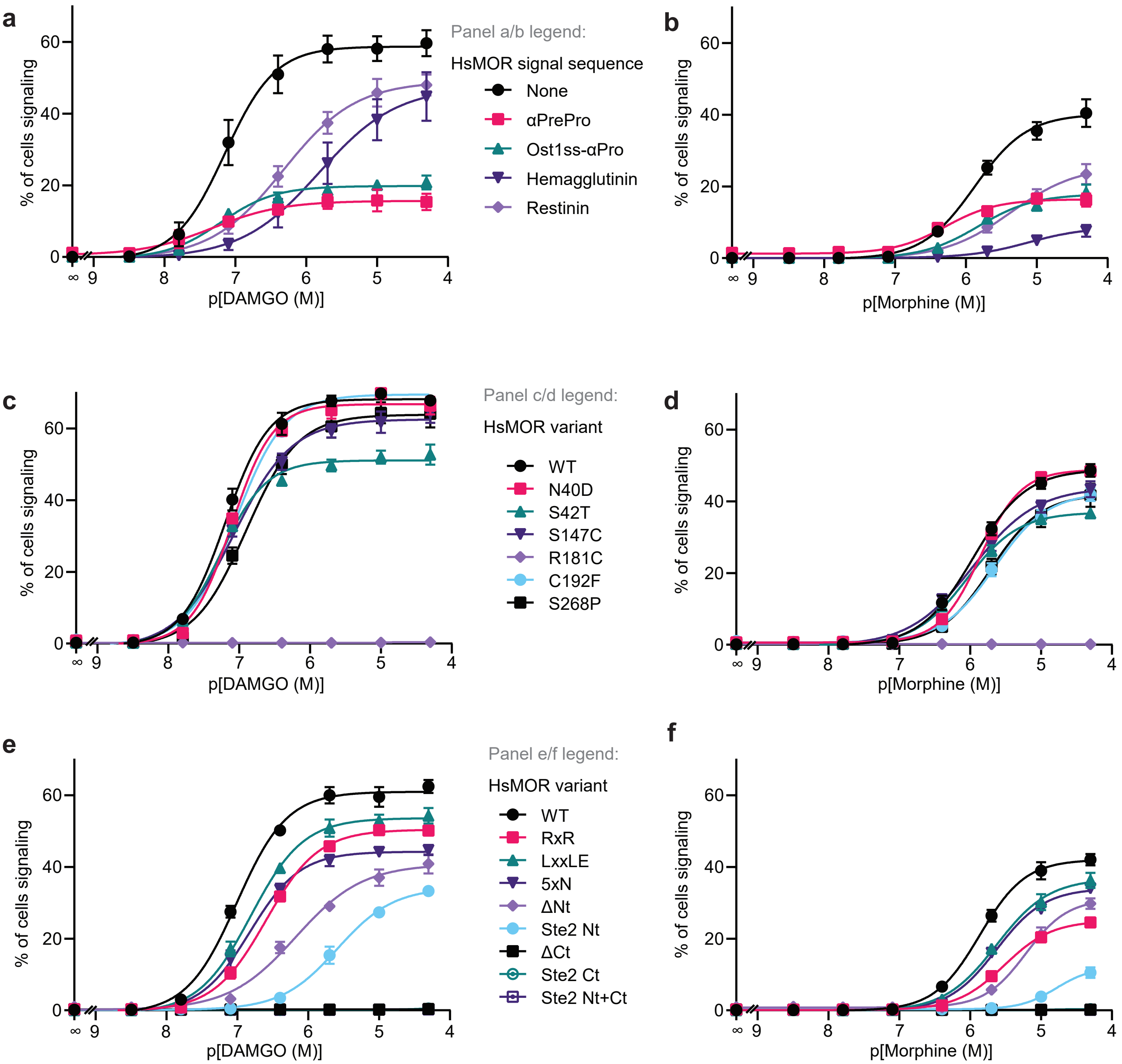

Supplementary Figure 3. DAMGO and morphine dose-response curves of biosensors based on introducing HsMOR variants into the cholesterol-producing chassis. **(a/b)** Curves of HsMOR signal sequence variants. n=3 biologically independent experiments, >7595 cells/strain/replicate. **(c/d)** Curves of HsMOR missense variants. n=3 biologically independent experiments, >8739 cells/strain/replicate. **(e/f)** Curves of HsMOR variants with mutations in terminal domains. n=3 biologically independent experiments, >7464 cells/strain/replicate. Data presented as mean +/- SEM. Source data are provided as a Source Data file.

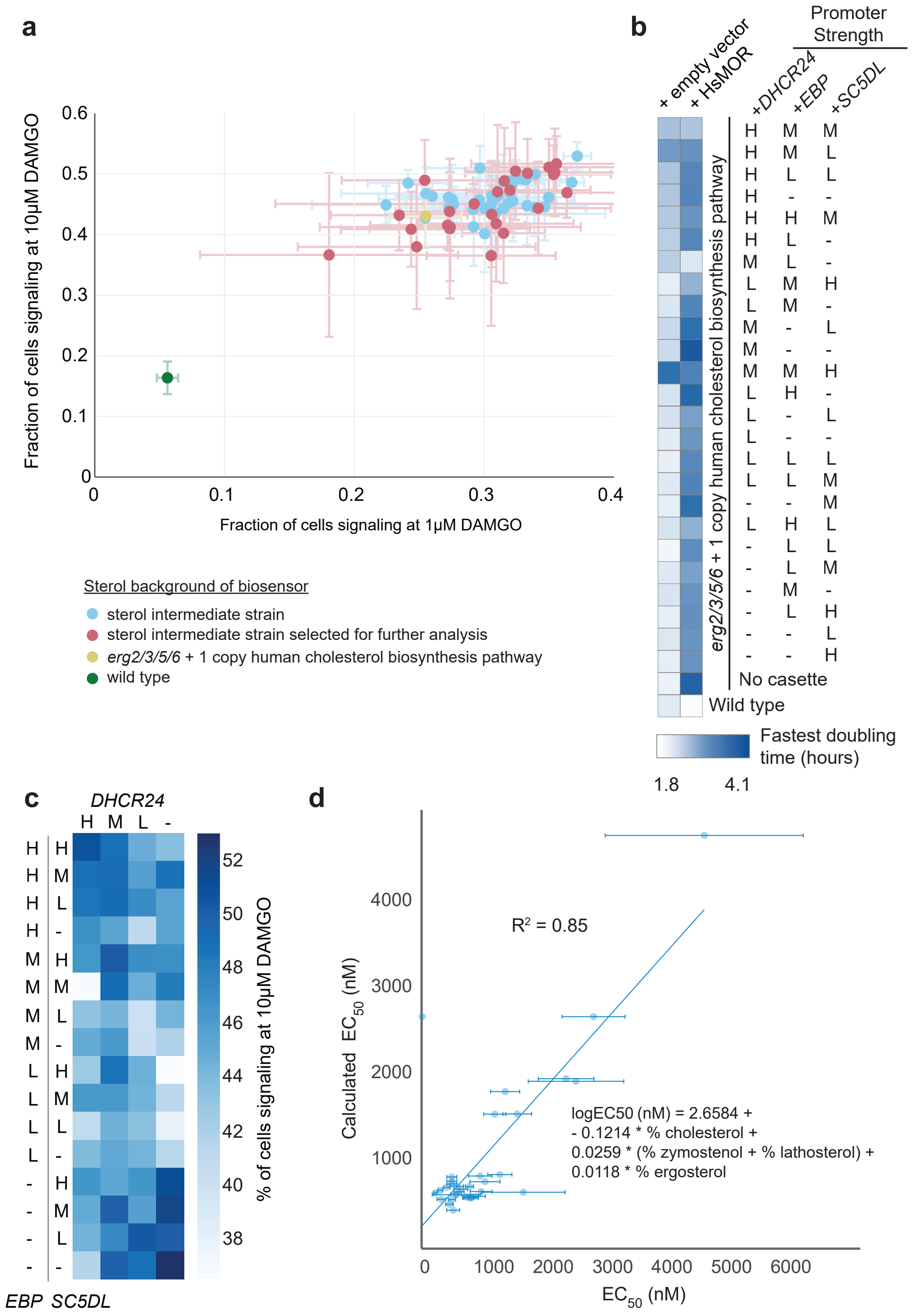

Supplementary Figure 4. Characteristics of cholesterol biosynthesis intermediate screen strains. **(a)** Percent of cells signaling with 10 μM and 1 μM DAMGO determined by flow cytometry. **(b)** Fastest doubling times of the strains chosen for further analysis. Promoter strength is indicated as high (H), medium (M), or low (L), with a dash denoting the absence of the gene. n=3 biologically independent experiments. **(c)** The percent of cells signaling at 10 μM DAMGO in the sterol intermediate screen. Unpaired one-way ANOVA, P = 3.626e-14: n=4 or 3 biologically independent experiments, 10000 cells/strain/replicate; P <1e-05 for all Dunnett's tests against the wild type sterol background. Results are means across strains with varying (inactive) DHCR7 status confirmed by failure of a Wilcoxon signed-rank test (P > 0.05) comparing the strong *DHCR7* expression and no gene conditions. **(d)** The measured EC50s and sterol profiles of the shortlisted sterol intermediate strains were related using a linear regression. EC50s calculated based on sterol intermediate percentages with this regression were plotted against measured EC50s. n=3 or 4 biologically independent experiments, 10000 cells/strain/replicate. Error bars represent standard deviation. Source data are provided as a Source Data file.

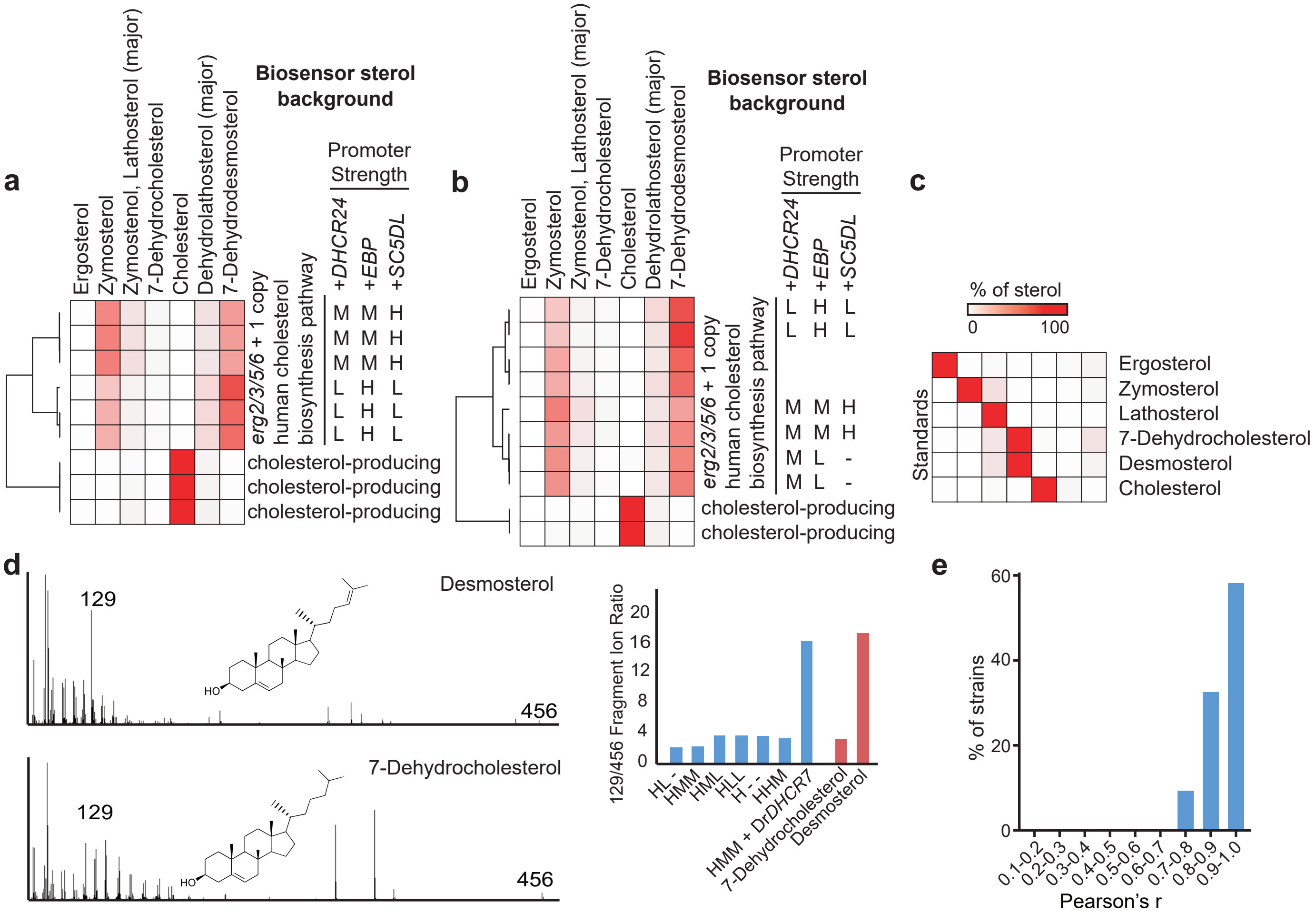

Supplementary Figure 5. Consistency of sterol proportions across biological and technical replicates. Heatmaps representing percentages of total sterol content across **(a)** technical and **(b)** biological replicates. Promoter strength is indicated as high (H), medium (M), or low (L), with a dash denoting the absence of the gene. Percentage of total sterols were calculated as described in the Methods. Technical replicates constitute independent injections while biological replicates are samples from different colonies. **(c)** Heatmaps depicting percent of sterols present in standards as measured in **a** and **b**. **(d)** MS spectra for intermediate sterols with the same m/z ratio are distinguishable by characteristic peaks. (Left) Unlike desmosterol, 7-dehydrocholesterol lacks a strong peak at 129 m/z. (Right) Fragment ion ratios of 129 to 456 m/z across standards 7-dehydrocholesterol, desmosterol and engineered yeast strains. Low 129/456 ratios are indicative of 7-dehydrocholesterol while higher ratios correspond to desmosterol. Strains are all in the biosensor background with one copy of the human cholesterol biosynthetic pathway integrated. Names reflect promoter strengths for additional DHCR24, EBP, and SC5DL copies. **(e)** Distribution of Pearson r correlation coefficients between sterol composition measured from 43 strains grown either 8 h or 48 h. Source data are provided as a Source Data file.

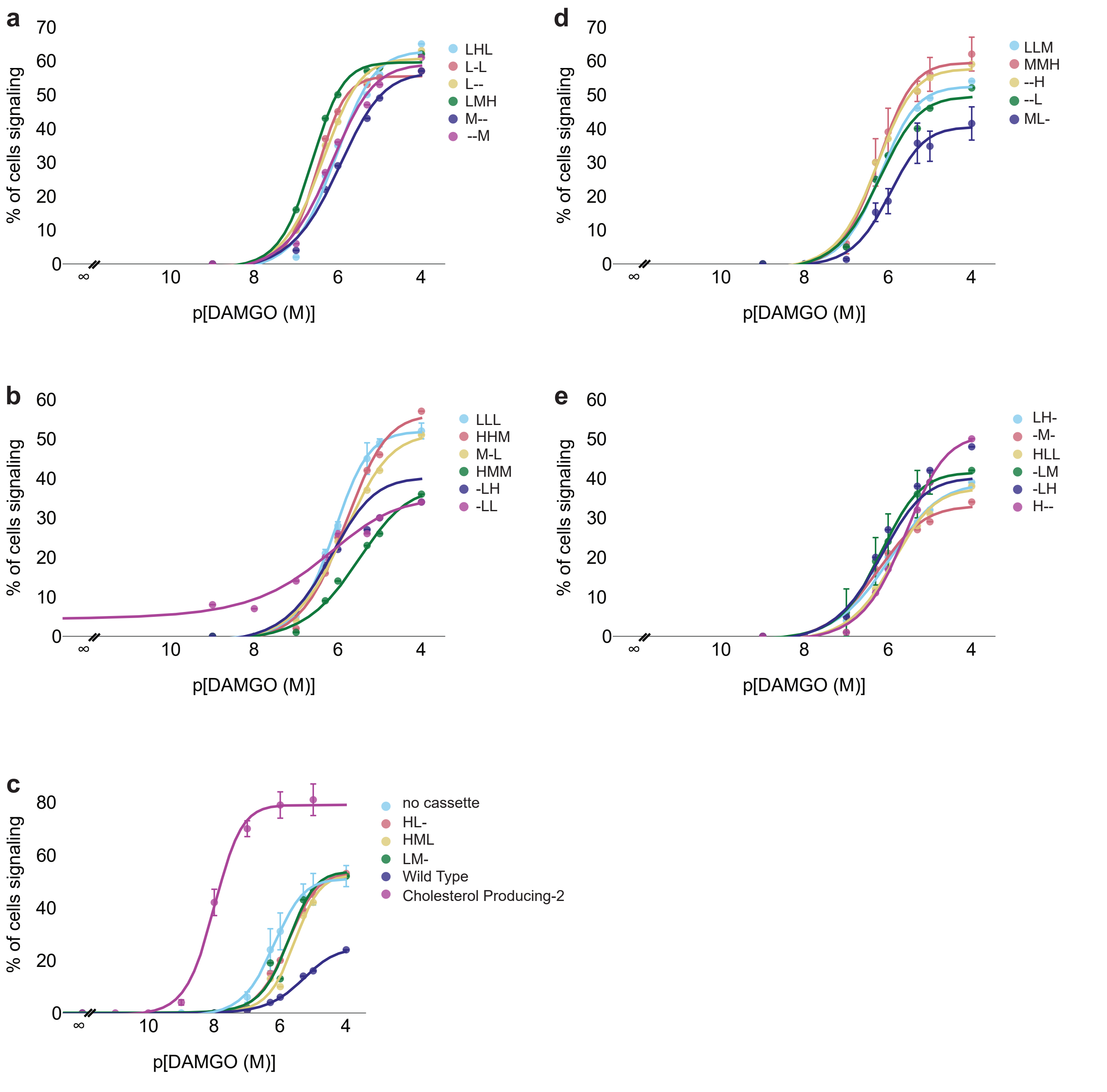

Supplementary Figure 6. TDose-response curves of HsMOR in sterol variant strains. **(a-e)** Curves of HsMOR in sterol variant strains. n=2, 3 or 4 biologically independent experiments, 10,000 cells/strain/replicate. Data are presented as mean values +/- SD when applicable. Source data are provided as a Source Data file.

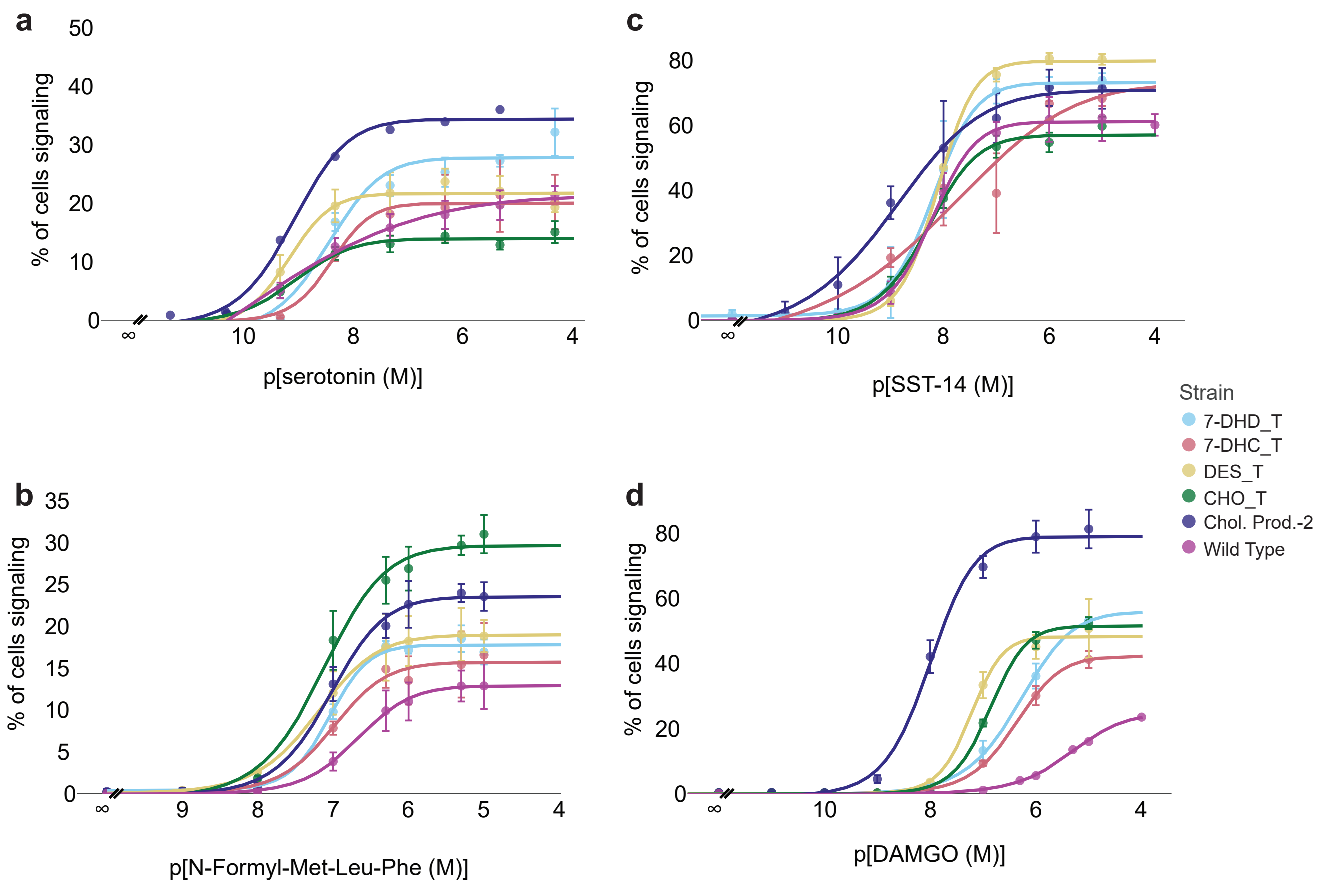

Supplementary Figure 7. Dose-response curves of HsMOR and non-opioid receptors in sterol variant strains. **(a)** Curves of HTR4B in sterol variant strains. n=3 biologically independent experiments, 10,000 cells/strain/replicate. N=2 for Chol. Prod.-2. **(b)** Curves of FPR1 in sterol variant strains. n=3 biologically independent experiments, 10,000 cells/strain/replicate. **(c)** Curves of SSTR5 in sterol variant strains. n=3 biologically independent experiments, 10,000 cells/strain/replicate. **(d)** Curves of HsMOR in sterol variant strains. n=3 biologically independent experiments, 10,000 cells/strain/replicate. N=2 for Wild Type. Data are presented as mean values +/- SD when applicable. Source data are provided as a Source Data file.
