# Supplementary material for: Functional expression of opioid receptors and other human GPCRs in yeast engineered to produce human sterols
Source: Nat Commun. 2022 May 24;13:2882. doi: 10.1038/s41467-022-30570-7 (PMC9130329; doi:10.1038/s41467-022-30570-7)
Supplement: Supplementary file 2 — Reporting Summary [file 41467_2022_30570_MOESM2_ESM.pdf]

nature portfolio

Corresponding author(s): Vincent JJ Martin

Last updated by author(s): May 3, 2022

Reporting Summary

Nature Portfolio wishes to improve the reproducibility of the work that we publish. This form provides structure for consistency and transparency in reporting. For further information on Nature Portfolio policies, see our Editorial Policies and the Editorial Policy Checklist.

Statistics

For all statistical analyses, confirm that the following items are present in the figure legend, table legend, main text, or Methods section.

n/a	Confirmed	
☐	☒	The exact sample size (*n*) for each experimental group/condition, given as a discrete number and unit of measurement
☐	☒	A statement on whether measurements were taken from distinct samples or whether the same sample was measured repeatedly
☐	☒	The statistical test(s) used AND whether they are one- or two-sided<br>*Only common tests should be described solely by name; describe more complex techniques in the Methods section.*
☒	☐	A description of all covariates tested
☐	☒	A description of any assumptions or corrections, such as tests of normality and adjustment for multiple comparisons
☐	☒	A full description of the statistical parameters including central tendency (e.g. means) or other basic estimates (e.g. regression coefficient) AND variation (e.g. standard deviation) or associated estimates of uncertainty (e.g. confidence intervals)
☐	☒	For null hypothesis testing, the test statistic (e.g. *F*, *t*, *r*) with confidence intervals, effect sizes, degrees of freedom and *P* value noted<br>*Give P values as exact values whenever suitable.*
☒	☐	For Bayesian analysis, information on the choice of priors and Markov chain Monte Carlo settings
☒	☐	For hierarchical and complex designs, identification of the appropriate level for tests and full reporting of outcomes
☐	☒	Estimates of effect sizes (e.g. Cohen's *d*, Pearson's *r*), indicating how they were calculated

Our web collection on statistics for biologists contains articles on many of the points above.

Software and code

Policy information about availability of computer code

Data collection	CFlow Plus 1.0.227.4 (flow cytometry), Volocity 5.5.1 (microscopy), CLARIOstar 5.21.R4 (plate reader)
Data analysis	MassHunter Workstation Software B.06.00, Fiji 1.51 23, Photoshop 2015.0.0, Illustrator 19.0.0, Excel 16.0.14326.20908, Prism 8.4.3

For manuscripts utilizing custom algorithms or software that are central to the research but not yet described in published literature, software must be made available to editors and reviewers. We strongly encourage code deposition in a community repository (e.g. GitHub). See the Nature Portfolio guidelines for submitting code & software for further information.

Data

Policy information about availability of data

All manuscripts must include a data availability statement. This statement should provide the following information, where applicable:

- Accession codes, unique identifiers, or web links for publicly available datasets
- A description of any restrictions on data availability
- For clinical datasets or third party data, please ensure that the statement adheres to our policy

Source data referenced within this manuscript is available in a Source Data file and raw acquisition files are available upon reasonable request.

Field-specific reporting

Life sciences study design

All studies must disclose on these points even when the disclosure is negative.

Sample size	Sample size calculations were not performed as we followed generally accepted standards such as usinig ~10,000 events for per flow cytometry sample, similar to past studies such as one by Lú Chau (referenced in the manuscript)..
Data exclusions	No data was excluded from analyses.
Replication	Unless otherwise noted, experiments were conducted using at least three independent biological replicates and all replicates were successful.
Randomization	Randomization was not necessary with our types of experimental designs as treatment and control groups were taken from the same single homogenous population.
Blinding	Blinding was not used in this work. Blinding was not used because most experiments produced results that could be batch analyzed with an explicit analytical pipeline giving little opportunity for experimenter bias to be introduced.

Reporting for specific materials, systems and methods

We require information from authors about some types of materials, experimental systems and methods used in many studies. Here, indicate whether each material, system or method listed is relevant to your study. If you are not sure if a list item applies to your research, read the appropriate section before selecting a response.

Materials & experimental systems

n/a	Involved in the study	
☒	☐	Antibodies
☒	☐	Eukaryotic cell lines
☒	☐	Palaeontology and archaeology
☒	☐	Animals and other organisms
☒	☐	Human research participants
☒	☐	Clinical data
☒	☐	Dual use research of concern

Methods

n/a	Involved in the study	
☒	☐	ChIP-seq
☐	☒	Flow cytometry
☒	☐	MRI-based neuroimaging

Flow Cytometry

Plots

Confirm that:

☐ The axis labels state the marker and fluorochrome used (e.g. CD4-FITC).

☐ The axis scales are clearly visible. Include numbers along axes only for bottom left plot of group (a 'group' is an analysis of identical markers).

☐ All plots are contour plots with outliers or pseudocolor plots.

☒ A numerical value for number of cells or percentage (with statistics) is provided.

Methodology

Sample preparation	Log phase yeast were assayed in media with the indicated agonists
Instrument	BD Accuri C6
Software	CFlow Plus 1.0.227.4 was used to acquire flow data.
Cell population abundance	Cells were not sorted, entire population was measured
Gating strategy	Cells were not initially gated. Cells were designated as "On" when they exceeded the fluorescence of 99-99.9% of the associated "Off" population (without an agonist present). Though the cell population was not gated, we provide an overview of our analysis strategy in Supplementary Information 1c

☒ Tick this box to confirm that a figure exemplifying the gating strategy is provided in the Supplementary Information.
