# Supplementary material for: Functional expression of opioid receptors and other human GPCRs in yeast engineered to produce human sterols
Source: Nat Commun. 2022 May 24;13:2882. doi: 10.1038/s41467-022-30570-7 (PMC9130329; doi:10.1038/s41467-022-30570-7)
Supplement: Supplementary file 10 — Description of Additional Supplementary Files [file 41467_2022_30570_MOESM10_ESM.pdf]

Title: Supplementary Data 1.
Description: A list of the yeast strains used in this study, including their strain IDs and sources.

Title: Supplementary Data 2.
Description: A list of all plasmids used in this study, including descriptions, IDs , and sources.

Title: Supplementary Data 3.
Description: The DNA sequences used for CRISPR-Cas9-based genomic edits including protospacer sequences and repair templates.

Title: Supplementary Data 4.
Description: All gene and tag DNA sequences used in this work.

Title: Supplementary Data 5.
Description: Summary of sensitivities of tested GPCRs in more native environments previously reported in the literature.

Title: Supplementary Data 6.
Description: Summary of different metrics of activity, including raw fluorescence values, for all biosensors used in this study.

Title: Supplementary Data 7.
Description: A list of all GPCR effectors used, their peptide sequences if relevant, and their sources.
